# Connexin 43 Hemichannel Activity Promoted by Pro-Inflammatory Cytokines and High Glucose Alters Endothelial Cell Function

**DOI:** 10.3389/fimmu.2018.01899

**Published:** 2018-08-15

**Authors:** Juan C. Sáez, Susana Contreras-Duarte, Gonzalo I. Gómez, Valeria C. Labra, Cristian A. Santibañez, Rosario Gajardo-Gómez, Beatriz C. Avendaño, Esteban F. Díaz, Trinidad D. Montero, Victoria Velarde, Juan A. Orellana

**Affiliations:** ^1^Departamento de Fisiología, Pontificia Universidad Católica de Chile, Santiago de Chile, Chile; ^2^Instituto de Neurociencias, Centro Interdisciplinario de Neurociencias de Valparaíso, Universidad de Valparaíso, Valparaíso, Chile; ^3^Departamento de Ginecología y Obstetricia, Escuela de Medicina, Facultad de Medicina, Pontificia Universidad Católica de Chile, Santiago, Chile; ^4^Departamento de Neurología, Escuela de Medicina and Centro Interdisciplinario de Neurociencias, Facultad de Medicina, Pontificia Universidad Católica de Chile, Santiago, Chile

**Keywords:** connexins, endothelium, inflammation, cytokines, gap junctions

## Abstract

The present work was done to elucidate whether hemichannels of a cell line derived from endothelial cells are affected by pro-inflammatory conditions (high glucose and IL-1β/TNF-α) known to lead to vascular dysfunction. We used EAhy 926 cells treated with high glucose and IL-1β/TNF-α. The hemichannel activity was evaluated with the dye uptake method and was abrogated with selective inhibitors or knocking down of hemichannel protein subunits with siRNA. Western blot analysis, cell surface biotinylation, and confocal microscopy were used to evaluate total and plasma membrane amounts of specific proteins and their cellular distribution, respectively. Changes in intracellular Ca^2+^ and nitric oxide (NO) signals were estimated by measuring FURA-2 and DAF-FM probes, respectively. High glucose concentration was found to elevate dye uptake, a response that was enhanced by IL-1β/TNF-α. High glucose plus IL-1β/TNF-α-induced dye uptake was abrogated by connexin 43 (Cx43) but not pannexin1 knockdown. Furthermore, Cx43 hemichannel activity was associated with enhanced ATP release and activation of p38 MAPK, inducible NO synthase, COX_2_, PGE_2_ receptor EP_1_, and P2X_7_/P2Y_1_ receptors. Inhibition of the above pathways prevented completely the increase in Cx43 hemichannel activity of cells treated high glucose and IL-1β/TNF-α. Both synthetic and endogenous cannabinoids (CBs) also prevented the increment in Cx43 hemichannel opening, as well as the subsequent generation and release of ATP and NO induced by pro-inflammatory conditions. The counteracting action of CBs also was extended to other endothelial alterations evoked by IL-1β/TNF-α and high glucose, including increased ATP-dependent Ca^2+^ dynamics and insulin-induced NO production. Finally, inhibition of Cx43 hemichannels also prevented the ATP release from endothelial cells treated with IL-1β/TNF-α and high glucose. Therefore, we propose that reduction of hemichannel activity could represent a strategy against the activation of deleterious pathways that lead to endothelial dysfunction and possibly cell damage evoked by high glucose and pro-inflammatory conditions during cardiovascular diseases.

## Introduction

The endothelial cell lining of vessels walls plays central roles in regulating vascular homeostasis, such as the maintenance of vessel integrity, supply of oxygen and nutrients to underlying tissues and promotion of a well-balanced redox and immune environment (1). Under physiological conditions, endothelial cells maintain a proper interface barrier between blood and tissue; surveilling and combating possible perturbations of invading pathogens or endogenous threats in response to tissue damage (2). The endothelium rapidly reacts to acute damage by modulating blood flow, permeability, leukocyte infiltration, and tissue edema (2). If the stimulus persists, chronic endothelial activation accompanied of sustained inflammation may lead to vascular dysfunction, precipitating macrophage recruitment, angiogenesis, and subsequent loss of vascular homeostasis (3). Chronic activation and dysfunction of endothelial cells are common features and part of the underlying origin of myocardial infarction, diabetes, stroke, obesity, unstable angina, metabolic syndrome, and sudden cardiac death (4). Although diverse conditions are present during these diseases, including high blood glucose levels, insulin resistance, oxidative stress, and upregulated cytokine production (5), the full underlying mechanisms associated with endothelial activation and dysfunction are not fully understood.

In the last decade, several studies have established that hemichannels mediate the physiological release of different signaling molecules (e.g., ATP, glutamate, NAD^+^, and PGE_2_) that preserve the progression of multiple biological processes, including long-term synaptic transmission (6), vessel contractility (7), and glucose sensing (8), among others. Hemichannels result from the oligomerization of six connexin monomers around a central pore, which along with forming the building blocks of gap junction channels, also acts as a solitary or non-junctional channels in the plasma membrane (9). Hemichannels are permeable to ions and small molecules; constituting routes of exchange between intracellular and extracellular compartments (10). Under certain pathophysiological scenarios, rather than being beneficial, the prolonged opening of hemichannels contributes to disease progression by different ways, including the enhanced release of paracrine substances (e.g., ATP and glutamate), intracellular Ca^2+^ handling alterations, and ionic and osmotic imbalance (11). A cornerstone underlying this phenomenon rise from the overproduction of inflammatory mediators as result of impaired operation of the innate and adaptive immune system (12).

There are plenty of data pointing out the detrimental effects of inflammation on endothelial function (2) and hyperglycemia is one of the most emblematic pro-inflammatory condition during different cardiovascular diseases (13, 14). Indeed, animal and clinical studies have shown that hyperglycemia causes the systemic production of pro-inflammatory cytokines such as TNFα and lL-1β (15, 16), as well as endothelial dysfunction (5). Among other changes, high glucose concentration in concert with pro-inflammatory cytokines alters numerous intracellular signaling pathways in endothelial cells (17, 18), which consequently lead to reduced endothelial barrier function, compromised vascular tone regulation and insulin resistance (5). Although prior evidence has described that IL-1β/TNF-α or high glucose (25–45 mM) causes a prominent opening of hemichannels in diverse brain cell types (8, 19–23), whether high glucose concentration and/or pro-inflammatory cytokines can modulate hemichannel activity in endothelial cells remain poorly studied.

We hypothesize that high glucose concentration in combination IL-1β/TNF-α increase the hemichannel activity of endothelial cells, resulting in several cell alterations. Here, we show that high glucose concentration and IL-1β/TNF-α and increase the activity of endothelial connexin 43 (Cx43) hemichannels. Inhibition of these channels prevented the alterations of purinergic signaling, [Ca^2+^]_i_ signal dynamics, and nitric oxide (NO) production. Moreover, two endogenous cannabinoids (CBs): methanandamide (Meth) or 2-arachidonylglycerol (2-AG), as well as one synthetic CB: WIN 55,212-2 (WIN), prevent these events. In particular, they counteracted the persistent opening of endothelial Cx43 hemichannels mainly due to increase in the amount of Cx43 in the cell surface, which consequently prevented the manifestation of different endothelial alterations.

## Materials and Methods

### Reagents and Antibodies

The mimetic peptides Gap19 (KQIEIKKFK, intracellular loop domain of Cx43), Tat-L2 (YGRKKRRQRRRDGANVDMHLKQIEIKKFKYGIEEHGK, second intracellular loop domain of Cx43), and ^10^panx1 [WRQAAFVDSY, first extracellular loop domain of pannexin1 (Panx1)] were obtained from Genscript (NJ, USA). HEPES, water (W3500), Dulbecco’s Modified Eagle Medium (DMEM), A74003, MRS2179, brilliant blue G (BBG), oxidized ATP (oATP), ns-398, sc-19220, indometacin, L-N6, SB203580, Lucifer yellow (LY), Meth and 2-AG, WIN-55,212-2 (WIN), Cx43 polyclonal antibody, ethidium (Etd) bromide, and probenecid (Prob) were purchased from Sigma-Aldrich (St. Louis, MO, USA). Fetal bovine serum (FBS) was obtained from Hyclone (Logan, UT, USA). Penicillin, streptomycin, FURA-2AM, DAF-FM diacetate, diamidino-2-phenylindole (DAPI), BAPTA-AM, goat anti-mouse Alexa Fluor 488 were obtained from Invitrogen (Carlsbad, CA, USA). The CB1 receptor antagonist (SR1): SR-141716A and the CB2 receptor antagonist (SR2): SR-144528 were kindly provided by Sanofi-Aventis Recherche (Bagneux, France). Normal goat serum (NGS) was purchased from Zymed (San Francisco, CA, USA). Anti-Cx43 monoclonal antibody (610061) was obtained from BD Biosciences (Franklin Lakes, NJ, USA). IL-1β and TNF-α were obtained from Roche Diagnostics (Indianapolis, MI, USA). Horseradish peroxidase (HRP)-conjugated anti-rabbit IgG, Sulfo-NHS-SS-biotin, and NeutrAvidin immobilized on agarose beads were purchased from Pierce (Rockford, IL, USA).

### Cell Cultures

The human endothelial cell line EAhy 926 was kindly donated by Cora-Jean S. Edgell, University of North Carolina, Chapel Hill. ECs were seeded onto plastic dishes (Nunclon) or onto glass coverslips (Gassalem, Limeil-Brevannes, France) in DMEM, supplemented with penicillin (5 U/ml), streptomycin (5 µg/mL), and 10% FBS and kept at 37°C in a 5% CO_2_/95% air atmosphere at nearly 100% relative humidity. Passaging was performed at ~90% confluence and cells were re-seeded at 1 × 10^4^ cells/cm^2^. Primary endothelial cells were isolated by collagenase (0.25 mg/mL) digestion from umbilical cord veins (HUVEC) from normal pregnancies and cultured (37°C, 5% CO_2_) up to passage 2 in medium 199 (M199) containing 10% new born calf serum, 10% fetal calf serum, 3.2 mM l-glutamine, and 100 U/mL penicillin–streptomycin. Passaging was performed at ~90% confluence and cells were re-seeded at 1 × 10^4^ cells/cm^2^.

### Treatments

Cells were treated for 1, 24, 48, or 72 h with a mixture of IL-1β and TNF-α (10 ng/mL of each) plus different concentrations of glucose (5, 25, or 45 mM). Mimetic peptides against Cx43 hemichannels (gap19 and Tat-L2, 100 µM) and Panx1 channels (^10^panx1, 100 µM), as well as Prob (500 µM), were applied to cell cultures 15 min prior to and co-applied with Etd for time-lapse recordings (see below). CB agonists: WIN, Meth, and 2-AG were applied 1 h prior to and co-applied with the cytokines and glucose treatment. SR1 and SR2 antagonists were co-applied with the CB agonists. Similarly, in another set of experiments, SB203580 (p38 MAP kinase inhibitor), L-N6 [inducible NO synthase (iNOS) inhibitor], indomethacin (COX_1_ and COX_2_ inhibitor), sc-560 (COX_1_ inhibitor), ns-398 (COX_2_ inhibitor), sc-19220 (EP_1_ receptor antagonist), BAPTA-AM (intracellular Ca^2+^ chelator), BBG (non-competitive P2X_7_ antagonist), oATP (P2X_7_ antagonist), MRS2179 (P2Y_1_ antagonist), or A740003 (P2X_7_ antagonist) were applied 1 h prior to and co-applied with IL-1β and TNF-α plus 25 mM glucose for 72 h.

### siRNA Transfection

siRNA duplexes against mouse Cx43 or Panx1 were predesigned and obtained from Origene (Rockville, MD, USA). siRNA (10 nM) was transfected using Oligofectamine (Invitrogen) according to the Origene application guide for Trilencer-27 siRNA. Sequences for siRNAs against human Cx43 and Panx1 were siRNA-Cx43: rGrCrCrTrTrCrTrTrGrCrTrGrArTrCrCrArGrTrGrGrTrArCrATC and siRNA-Panx1: rGrArTrCrTrCrGrArTrTrGrGrTrArCrArCrArGrArTrArArGrCTG, respectively. Transfection experiment was performed 24 h before treating cells with IL-1β and TNF-α plus 25 mM glucose for 72 h.

### Dye Uptake and Time-Lapse Fluorescence Imaging

For time-lapse fluorescence imaging, cells plated on glass coverslips were washed twice in Hank’s balanced salt solution. Then, cells were incubated with Locke’s solution containing 5 µM Etd and mounted on the stage of an Olympus BX 51W1I upright microscope with a 40× water immersion objective for time-lapse imaging. Images were captured by a Retiga 1300I fast-cooled monochromatic digital camera (12-bit) (Qimaging, Burnaby, BC, Canada) controlled by imaging software Metafluor software (Universal Imaging, Downingtown, PA, USA) every 30 s (exposure time = 0.5 s; excitation and emission wavelengths were 528 and 598 nm, respectively). The fluorescence intensity recorded from 25 regions of interest (representing 25 cells per coverslip) was defined as the subtraction (F-F0) between the fluorescence (F) from respective cell (25 cells per field) and the background fluorescence (F0) measured where no labeled cells were detected. The mean slope of the relationship F-F0 over a given time interval (ΔF/ΔT; F0 remained constant along the recording time) represents the Etd uptake rate. To assess for changes in slope, regression lines were fitted to points before and after the various experimental conditions using Excel software, and mean values of slopes were compared using GraphPad Prism software and expressed as AU/min. At least four replicates (four sister coverslips) were measured in each independent experiment.

### Western Blot Analysis

Cells were rinsed twice with PBS (pH 7.4) and harvested by scraping with a rubber policeman in ice-cold PBS containing 5 mM EDTA, Halt (78440), and M-PER protein extraction cocktail (78501) according to the manufacturer instructions (Pierce, Rockford, IL, USA). The cell suspension was sonicated on ice. Proteins were measured using the Bio-Rad Bradford assay. Aliquots of cell lysates (100 µg of protein) were resuspended in Laemmli’s sample buffer, separated in an 8% sodium dodecyl sulfate polyacrylamide gel electrophoresis (SDS-PAGE) and electro-transferred to nitrocellulose sheets. Nonspecific protein binding was blocked by incubation of nitrocellulose sheets in PBS-BLOTTO (5% nonfat milk in PBS) for 30 min. Blots were then incubated with primary antibody at 4°C overnight, followed by four 15 min washes with PBS. Then, blots were incubated with HRP-conjugated goat anti-rabbit antibody at room temperature for 1 h and then rinsed four times with PBS for 15 min. Immunoreactivity was detected by enhanced chemiluminescence reaction using the SuperSignal kit (Pierce, Rockford, IL, USA) according to the manufacturer’s instructions.

### Cell Surface Biotinylation and Quantification

Cells cultured on 100-mm dishes were washed three times with ice-cold Hank’s saline solution (pH 8.0), and 3 mL of sulfo-NHS-SS-biotin solution (0.5 mg/mL) was added followed by a 30 min incubation at 4°C. Then, cells were washed three times with ice-cold saline containing 15 mM glycine (pH 8.0) to block unreacted biotin. The cells were harvested and incubated with an excess of immobilized NeutrAvidin (1 mL of NeutrAvidin per 3 mg of biotinylated protein) for 1 h at 4°C after which 1 mL of wash buffer (saline solution, pH 7.2 containing 0.1% SDS and 1% Nonidet P-40) was added. The mixture was centrifuged for 2 min at 600 *g* at 4°C. The supernatant was removed and discarded, and the pellet was resuspended in 40 µL of saline solution, pH 2.8 containing 0.1 M glycine, to release the proteins from the biotin. After the mixture was centrifuged at 600 *g* at 4°C for 2 min, the supernatant was collected, and the pH was adjusted immediately by adding 10 µL of 1 M Tris, pH 7.5. Relative protein amount was measured using Western blot analysis as described above. Resulting immunoblot signals were scanned, and the densitometric analysis was performed with IMAGEJ software.

### Dye Coupling

Cells plated on glass coverslips were bathed with recording medium (${\text{HCO}}_{\text{3}}^{-}$ free F-12 medium buffered with 10 mM HEPES, pH 7.2) and permeability mediated by gap junctions was tested by evaluating the transfer of LY to neighboring cells. Briefly, single ECs were iontophoretically microinjected with a glass micropipette filled with 75 mM LY (5% w/v in 150 mM LiCl) in recording medium containing 200 µM La^3+^ to avoid cell leakage of the microinjected dye *via* hemichannels, leading to underscore the extent of dye coupling. Fluorescent cells were observed using a Nikon inverted microscope equipped with epifluorescence illumination (Xenon arc lamp) and Nikon B filter to LY (excitation wavelength 450–490 nm; emission wavelength above 520 nm) and XF34 filter to DiI fluorescence (Omega Optical, Inc., Brattleboro, VT, USA). Photomicrographs were obtained using a CCD monochrome camera (CFW-1310M; Scion; Frederick, MD, USA). Three minutes after dye injection, cells were observed to determine whether dye transfer occurred. The incidence of dye coupling was scored as the percentage of injections that resulted in dye transfer from the injected cell to more than one neighboring cell. Three experiments were performed for every treatment and dye coupling was tested by microinjecting a minimum of 10 cells per experiment.

### Immunofluorescence

Cells grown on glass coverslips were fixed at room temperature with 2% paraformaldehyde for 30 min and then washed three times with PBS. Then, cells were incubated three times for 5 min in 0.1 M PBS-glycine, followed by 30 min incubation with 0.1% PBS-Triton X-100 containing 10% NGS. The permeabilized cells were incubated with anti-β-tubulin monoclonal antibody (Sigma, 1:400) and anti-Cx43 polyclonal antibody (SIGMA, 1:400) diluted in 0.1% PBS-Triton X-100 with 2% NGS at 4°C overnight. After five rinses in 0.1% PBS-Triton X-100, cells were incubated with goat anti-mouse IgG Alexa Fluor 555 (1:1,000), goat anti-rabbit IgG Alexa Fluor 488 (1:1,000), or Alexa Fluor 488-phalloidin at room temperature for 50 min. After several rinses, coverslips were mounted in DAPI Fluoromount-G medium and examined with an Olympus BX 51W1I upright microscope with a 40× water immersion objective or a confocal laser-scanning microscope with a 63× objective (Olympus, Fluoview FV1000, Tokyo, Japan).

### Intracellular Ca^2+^ and NO Imaging

Cells plated on glass coverslips were loaded with 5 µM Fura-2-AM or 5 µM DAF-FM diacetate in DMEM without serum at 37°C for 45 min and then washed three times in Locke’s solution (154 mM NaCl, 5.4 mM KCl, 2.3 mM CaCl_2_, 5 mM HEPES, pH 7.4) followed by de-esterification at 37°C for 15 min. The experimental protocol for Ca^2+^ signal and NO imaging involved data acquisition every 5 s (emission at 510 and 515 nm, respectively) at 340/380-nm and 495 excitation wavelengths, respectively, using an Olympus BX 51W1I upright microscope with a 40× water immersion objective. Changes were monitored using an imaging system equipped with a Retga 1300I fast-cooled monochromatic digital camera (12-bit) (Qimaging, Burnaby, BC, Canada), monochromator for fluorophore excitation, and METAFLUOR software (Universal Imaging, Downingtown, PA, USA) for image acquisition and analysis. Analysis involved determination of pixels assigned to each cell. The average pixel value allocated to each cell was obtained with excitation at each wavelength and corrected for background. Due to the low excitation intensity, no bleaching was observed even when cells were illuminated for a few minutes. The FURA-2 ratio was obtained after dividing the 340-nm by the 380-nm fluorescence image on a pixel-by-pixel base (*R* = *F*_340 nm_/*F*_380 nm_).

### Measurement of Extracellular ATP Concentration

Cells were seeded (2 × 10^6^ cells in 35 mm dishes) in DMEM containing 10% FBS and treated with IL-1β and TNF-α plus 25 mM glucose for 72 h. Supernatants were collected, filtered (0.22 µm), and stored at −20°C before used for experiments. Then, extracellular ATP was measured using a luciferin/luciferase bioluminescence assay kit (Sigma-Aldrich). The amount of ATP in each sample was inferred from standard curves and normalized for the protein concentration as determined by the BCA assay (Pierce).

### Data Analysis and Statistics

For each data group, results were expressed as mean ± SEM; *n* refers to the number of independent experiments. Detailed statistical results were included in the figure legends. Statistical analyses were performed using GraphPad Prism (version 7, GraphPad Software, La Jolla, CA, USA). Normality and equal variances were assessed by the Shapiro–Wilk normality test and Brown–Forsythe test, respectively. Unless otherwise stated, data that passed these tests were analyzed by unpaired *t* test in case of comparing two groups, whereas in case of multiple comparisons, data were analyzed by one or two-way analysis of variance (ANOVA) followed, in case of significance, by a Tukey’s *post hoc* test. A probability of *p* < 0.05 was considered statistically significant.

## Results

### IL-1β/TNF-α Plus High Glucose Enhance the Activity of Cx43 Hemichannels in Endothelial Cells

Previous studies have revealed that stimulation with IL-1β/TNF-α or high glucose (25–45 mM) causes a prominent opening of hemichannels in diverse brain cell types (8, 19–22). Given that inflammatory mediators play crucial roles in the activation of endothelial cells and because hemichannels may contribute to this process as they do in other tissues (24, 25), we examined whether two pro-inflammatory cytokines and high glucose could modulate the activity of these channels in the human endothelial cell line EAhy 926. The functional state of hemichannels was evaluated by measuring the rate of ethidium (Etd) uptake. Etd only move across the plasma membrane in normal cells by permeating specific large-pore channels such as hemichannels (26). After its binding to RNA and DNA, Etd becomes fluorescent, revealing channel opening when appropriate blockers are employed.

**Figure 1:**
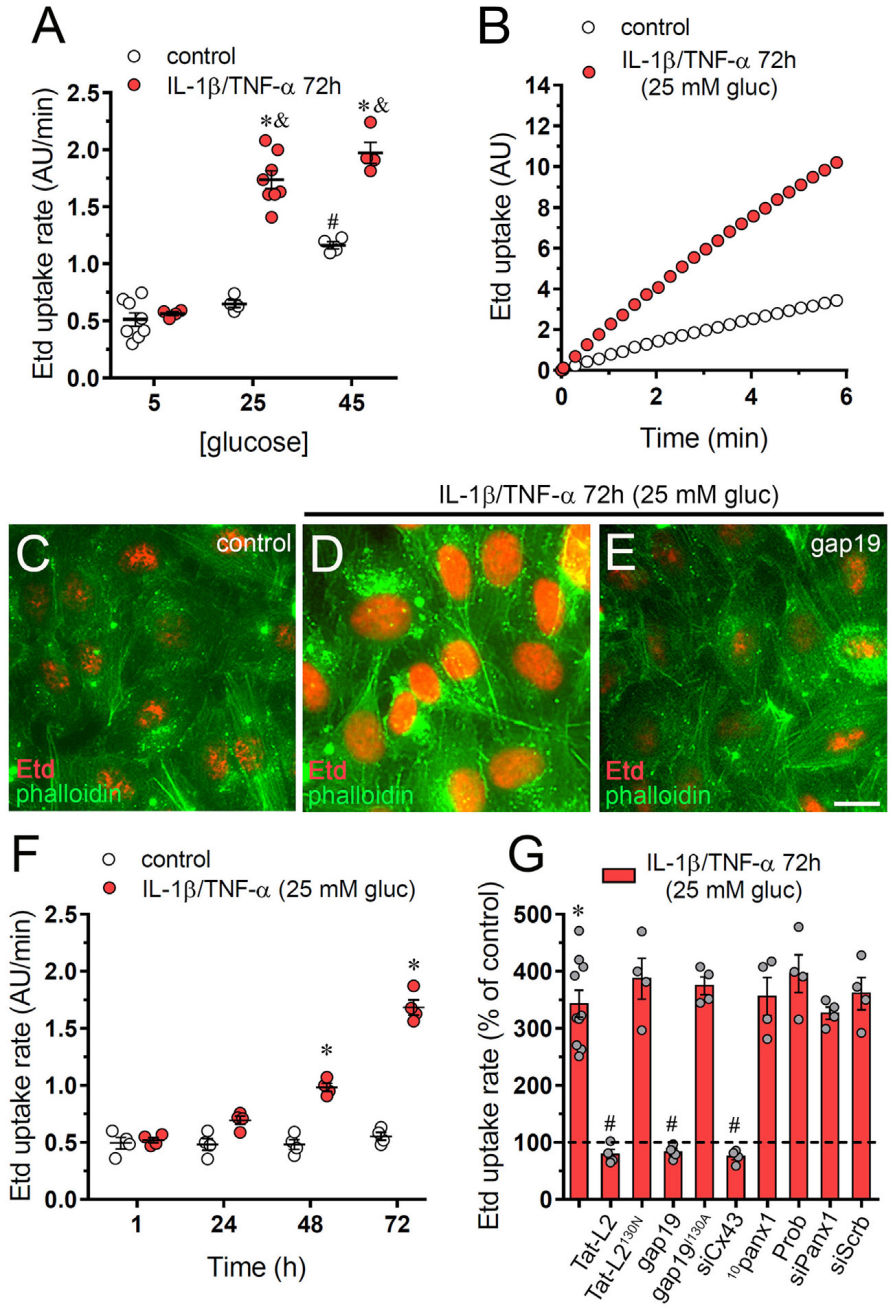
High glucose and IL-1β plus TNF-α increase the activity of connexin 43 (Cx43) hemichannels in endothelial cells. **(A)** Averaged Etd uptake rate of EAhy cells treated for 72 h with different concentrations of glucose alone (control, white circles) or in combination with a mixture of IL-1β/TNF-α (red circles; 10 ng/mL for each). ^#^*p* < 0.05, 45 mM glucose (control) compared to 5 mM glucose (control), *p < 0.05, IL-1β/TNF-α compared to control; ^&^*p* < 0.05, high glucose (IL-1β/TNF-α) compared to 5 mM glucose (IL-1β/TNF-α) [two-way analysis of variance (ANOVA) followed by Tukey’s *post hoc* test]. **(B)** Time-lapse measurements of Etd uptake by EAhy cells treated for 72 h with 5 mM glucose (control, white circles) or 25 mM glucose and IL-1β/TNF-α (red circles). **(C–E)** Representative immunofluorescence images depicting phalloidin-actin (green) and Etd-nucleus (red) staining from dye uptake measurements (10 min exposure to dye) in EAhy cells treated for 72 h with 5 mM glucose [control **(C)**], 25 mM glucose and IL-1β/TNF-α **(D)** alone or plus 100 µM gap19. **(F)** Averaged Etd uptake rate by EAhy cells treated for several time periods with 5 mM glucose (control, white circles) or 25 mM glucose and IL-1β/TNF-α (red circles). *p < 0.05, IL-1β/TNF-α and high glucose compared to control (two-way ANOVA followed by Tukey’s *post hoc* test). **(G)** Averaged Etd uptake rate normalized with control condition (dashed line) by EAhy cells treated for 72 h with 25 mM glucose and IL-1β/TNF-α alone or in combination with the following blockers: 100 µM Tat-L2, 100 µM Tat-L2^H126K/I130N^, 100 μM gap19, 100 µM gap19^I130A^, siRNA^Cx43^, 100 µM ^10^panx1, 500 µM probenecid (Prob), siRNA^Panx1^; and siRNA^scrb^. *p < 0.05, IL-1β/TNF-α and high glucose compared to control; ^#^*p* < 0.05, effect of blockers compared IL-1β/TNF-α and high glucose (one-way ANOVA followed by Tukey’s *post hoc* test). Data were obtained from at least three independent experiments (see scatter dot plot) with four repeats each one (≥35 cells analyzed for each repeat). Calibration bar = 20 µm.

After incubation with 45 mM but not 25 mM glucose, EAhy cells exhibited a significant twofold increment in Etd uptake compared with physiological glucose concentration (5 mM) (Figure 1A). Relevantly, a combination of IL-1β and TNF-α (10 ng/mL of each) enhanced the response evoked by 45 mM glucose (Figure 1A), whereas 25 mM glucose (hereinafter referred as to “high glucose”) only increased Etd uptake when applied in combination with IL-1β/TNF-α (Figures 1A–D). Moreover, IL-1β/TNF-α and high glucose triggered a time-dependent proportional rise in Etd uptake, being 72 h of treatment the most significant as it evoked a 3.5-fold augment in relation to control treatment (Figure 1F). No changes in Etd uptake were observed upon treatment with IL-1β/TNF-α and plus high sucrose or high mannitol excluding the possibility of an osmolarity-mediated response (Figure S1A in Supplementary Material).

Endothelial cells express functional Cx43 hemichannels (27–29) and Panx1 channels (30, 31). Pannexins encompass a three-member protein family that constitute unopposed membrane channels referred as pannexons that—just like hemichannels—allow paracrine/autocrine communication in cellular tissues (32). The involvement of Cx43 hemichannels in IL-1β/TNF-α and high glucose-mediated Etd uptake was examined employing specific mimetic peptides (Tat-L2 and gap19) with sequences homologous to intracellular L2 loop domains of Cx43 (33, 34). Cells treated with IL-1β/TNF-α and high glucose for 72 h and incubated for 15 min of incubation with Tat-L2 (100 µM) or gap19 (100 µM) before and during the dye uptake evaluation showed an Etd uptake close to that of control cells (Figures 1C,E,G). In addition, a mutated TAT-L2 (200 µM TAT-L2^H126K/I130N^), in which 2 aa crucial for interaction of L2 domain to the carboxyl tail of Cx43 are modified, was unsuccessful in trigger a comparable inhibition (Figure 1G). Likewise, we noticed that an inactive form of gap19 containing the I130A modification (gap19^I130A^), did not inhibit the IL-1β/TNF-α and high glucose-induced Etd uptake in EAhy cells (Figure 1G). Consistent with these findings, knockdown of Cx43 with siRNA fully abolished the Etd uptake caused by IL-1β/TNF-α and high glucose (Figure 1G). Conversely, scrambled siRNA, siRNA for Panx1, the Panx1 mimetic peptide ^10^panx1 (100 µM), or probenecid (200 µM) failed to cause a similar suppression (Figure 1G). These results strongly bring up that IL-1β/TNF-α and high glucose significantly increase the activity of Cx43 hemichannels, but not Panx1 channels in EAhy endothelial cells.

### Endothelial Cx43 Hemichannel Activity Induced by IL-1β/TNF-α Plus High Glucose Depends on p38 MAP Kinase/iNOS/COX_2_/EP_1_ and Purinergic Pathways

**Figure 2:**
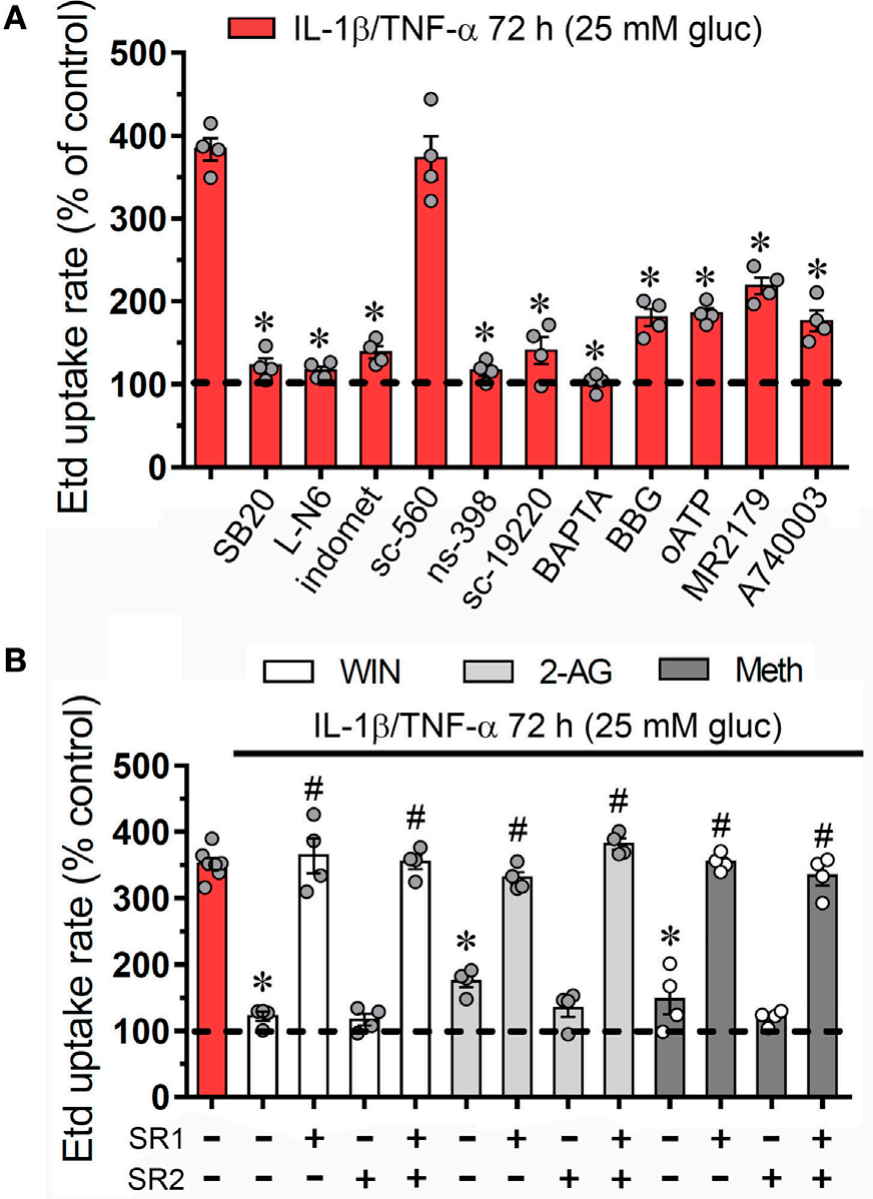
Endothelial connexin 43 (Cx43) hemichannel opening evoked by high glucose and IL-1β/TNF-α depends on Ca^2+^ signaling and activation of p38 MAPK/inducible NO synthase/COX_2_-dependent pathways and EP_1_/P2 receptors: prevention by cannabinoids. **(A)** Averaged Etd uptake rate normalized with control conditions (5 mM glucose, dashed line) of EAhy cells treated for 72 h with 25 mM glucose and IL-1β/TNF-α alone or in combination with the following agents: 10 µM SB203580, 1 µM L-N6, 15 µM indometacin (indomet); 1 µM sc-560; 5 µM ns-398; 20 µM sc-19220, 10 µM BAPTA, 10 µM Brilliant blue G (BBG), 200 µM oxidized ATP (oATP), 10 µM MRS2179; and 10 µM A740003. *p < 0.05, effect of blockers compared to IL-1β/TNF-α and high glucose [one-way analysis of variance (ANOVA) followed by Tukey’s *post hoc* test]. **(B)** Averaged Etd uptake rate normalized with control conditions (5 mM glucose, dashed line) by EAhy cells treated for 72 h with 25 mM glucose and IL-1β/TNF-α (red bar) alone or in combination with the following cannabinoids: WIN (5 µM, white bars), 2-arachidonylglycerol (5 µM, light gray bars), and Meth (5 µM, dark gray bars). It is also shown the effect of the respective cannabinoid co-treatment with the CB_1_ or CB_2_ receptor antagonist, SR-141716A (5 µM) and/or SR-144528 (5 µM), respectively. *p < 0.05, effect of each cannabinoid compared to the effect induced by 72 h treatment with IL-1β/TNF-α and high glucose; **^#^***p* < 0.05, effect of each cannabinoid receptor antagonist compared to the effect of the respective cannabinoid (one-way ANOVA followed by Tukey’s *post hoc* test). Data were obtained from at least three independent experiments (see scatter dot plot) with four repeats each one (≥35 cells analyzed for each repeat).

During inflammatory conditions, endothelial cells display a strong stimulation of the iNOS and cyclooxygenase 2 (COX_2_) (35, 36), two enzymes that generate byproducts (NO and prostaglandins, respectively) associated with Cx43 hemichannel activation (37–39). In addition, prior research has unveiled the participation of p38 MAP kinase (p38 MAPK) in both the opening of Cx43 hemichannels (20) and inflammatory activation of endothelial cells (40, 41). Accordingly, we examined the influence of p38 MAPK, iNOS, and COX_2_ pathways on the IL-1β/TNF-α and high glucose-induced Etd uptake in EAhy cells. The Etd uptake triggered by IL-1β/TNF-α and high glucose treatment for 72 h was greatly reduced by blockade of p38 MAPK with SB202190 (10 µM) or inhibition of iNOS with L-N6 (5 µM) (Figure 2A). Notably, COX inhibition by indomethacin (15 µM) reduced the ~3.5-fold increase on Etd uptake evoked by IL-1β/TNF-α and high glucose to control conditions (Figure 2A). To investigate which COX was implicated in the above effect, we used sc-560 and ns-398, specific inhibitors for COX_1_ and COX_2_, respectively. sc-560 (1 µM) failed in neutralizing the Etd uptake caused by IL-1β/TNF-α and high glucose, whereas ns-398 (5 µM) completely abolished it (Figure 2A).

Previous findings indicate that NO elevates COX_2_ activity and prostaglandin E_2_ (PEG_2_) generation in macrophages (42) and a similar phenomenon seems to occur in inflamed endothelial cells (43). Given that activation of PEG_2_ receptor 1 (EP_1_) lead to increases in [Ca^2+^]_i_ (44) and the latter is a well-known mechanism that increase the open probability of Cx43 hemichannels (45), we examined if this signaling is involved in the IL-1β/TNF-α and high glucose-mediated Etd uptake in EAhy cells. Blockade of EP_1_ receptor with sc-19220 (20 µM) was found to diminish the Etd uptake caused by IL-1β/TNF-α and high glucose, whereas 5 µM BAPTA-AM, a Ca^2+^ chelator, caused a similar inhibition (Figure 2A). The opening of Cx43 hemichannels has been correlated with [Ca^2+^]_i_-mediated purinergic signaling (46, 47), thereby, we tested if purinergic receptors participate in the IL-1β/TNF-α and high glucose-induced Etd uptake in EAhy cells. Remarkably, 200 µM oATP, a general P2X receptor blocker, or 10 µM A740003 and 10 µM BBG, both P2X_7_ receptor inhibitors, partially antagonized the Etd uptake induced by IL-1β/TNF-α and high glucose (Figure 2A). Similarly, the blockade of P2Y_1_ receptors with 10 µM MRS2179 evoked a partial but significant reduction in the IL-1β/TNF-α and high glucose-induced Etd uptake. All these data suggest that endothelial Cx43 hemichannel activity triggered by IL-1β/TNF-α and high glucose rely on the stimulation of p38 MAPK/iNOS/COX_2_/EP_1_–dependent pathway(s) and the P2X_7_/P2Y_1_ receptor-mediated changes in cytoplasmic Ca^2+^ signal.

### CBs Counteract the Opening of Cx43 Hemichannels Induced by IL-1β/TNF-α Plus High Glucose in Endothelial Cells

An unresolved topic in the field of hemichannels is to recognize compounds that could prevent their increased opening during pathological conditions. Possible aspirants for this intent are CBs, as they successfully prevent the persistent Cx43 hemichannel opening triggered by different inflammatory conditions in glial cells (48–50). Whether plant-derived, synthetic, or endocannabinoids, CBs are biolipid molecules that activate at least two CB receptors: CB_1_ and CB_2_ (51). Because endothelial cells express CB_1_/CB_2_ receptors (52) and CBs elicit anti-inflammatory defense facing cytokine-dependent endothelial dysfunction (53), we examined whether CBs could ameliorate the increase in Cx43 hemichannel activity evoked by IL-1β/TNF-α and high glucose in EAhy cells.

To elucidate whether CBs could regulate the Cx43 hemichannel activity in EAhy cells, we pre-incubated the cells with synthetic and endogenous CBs for 1 h and then were co-applied for 72 h along with IL-1β/TNF-α and high glucose. WIN-55,212-2 (WIN; 5 µM), a synthetic agonist of CB_1_/CB_2_ receptors, completely blunted the IL-1β/TNF-α and high glucose-induced Etd uptake in EAhy cells since these cells showed an Etd uptake comparable to control values (Figure 2B). Furthermore, we used two endogenous CB_1_/CB_2_ receptor agonists: 2-AG and methanandamide (Meth), the latter being a synthetic analog of the endocannabinoid anandamide. We observed that 5 µM 2-AG and 5 µM Meth reduced the increase in Etd uptake rate of EAhy cells caused by the IL-1β/TNF-α and high glucose treatment to ~169 and ~146%, respectively (Figure 2B). CB_1_ and CB_2_ receptor antagonists, SR-141716 (SR1) and SR-144528 (SR2), respectively, were employed to characterize the sub-type of CB receptor implicated in the counteracting response on Etd uptake evoked by IL-1β/TNF-α and high glucose (Figure 2B). With the application of 10 µM SR1 antagonist, WIN, 2-AG, and Meth failed in preventing the IL-1β/TNF-α and high glucose-mediated Etd uptake in EAhy cells, whereas 10 µM SR2 was ineffective in evoke a comparable preventing effect (Figure 2B). These findings indicate that CB_1_, but not CB_2_ receptors are the major contributors to the WIN, 2-AG, and Meth counteracting responses of the IL-1β/TNF-α and high glucose-evoked Cx43 hemichannel activity in endothelial cells.

### WIN Counteract the Increment in Cx43 Surface Amount and Gap Junctional Uncoupling Triggered by IL-1β/TNF-α Plus High Glucose in Endothelial Cells

**Figure 3:**
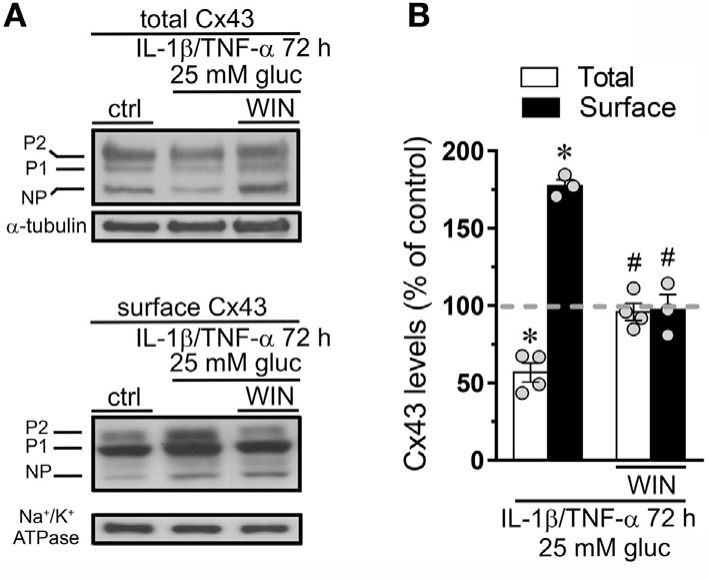
WIN counteracts the increase in surface levels of connexin 43 (Cx43) induced by high glucose and IL-1β/TNF-α in endothelial cells. **(A)** Total (upper panel) and surface (bottom panel) levels of Cx43 by EAhy cells treated for 72 h with 5 mM glucose (control), 25 mM glucose, and IL-1β/TNF-α alone or in combination with 5 µM WIN. The Cx43 phosphorylated (P1–P2) and non-phosphorylated (NP) forms are indicated in the left. Total and surface amount of each analyzed band were normalized according to the amount of α-tubulin and Na^+^/K^+^ ATPase detected in each lane, respectively. **(B)** Quantification of total (white bars) and surface (black bars) amount of Cx43 normalized to control (dashed line) in EAhy cells harvested 72 h after treatment with 25 mM glucose and IL-1β/TNF-α alone or in combination with 5 µM WIN. *p < 0.05, IL-1β/TNF-α and high glucose compared to control; ^#^*p* < 0.05, effect of each cannabinoid compared to the effect induced by IL-1β/TNF-α and high glucose (two-tailed Student’s unpaired *t*-test). Averaged data were obtained from at least three independent experiments (see scatter dot plot).

Connexin 43 hemichannel activity could depend on increments in both the open probability per channel and/or the number of channels at the cell surface. Previous studies have correlated the hemichannel-dependent Etd uptake with elevated surface levels of Cx43 in different cell types (8, 39, 50) or increase in open probability without changes in the total amount of Cx43 in the cell surface (54). Here, we evaluated if the counteracting effect of CBs on IL-1β/TNF-α and high glucose-mediated hemichannel activity depend on changes in surface amount of Cx43. IL-1β/TNF-α and high glucose caused a slight but significant ~30% decrease in total Cx43 compared to control conditions, a response fully suppressed by 5 µM WIN (Figures 3A,B). Moreover, IL-1β/TNF-α and high glucose also evoked a ~1.7-fold rise in surface amount of Cx43 and experiments with WIN fully blunted this effect (Figures 3A,B). Therefore, the ameliorative effects of CBs on IL-1β/TNF-α and high glucose-induced Cx43 hemichannel activity may take place because they interfere with the increment in surface amount of Cx43.

**Figure 4:**
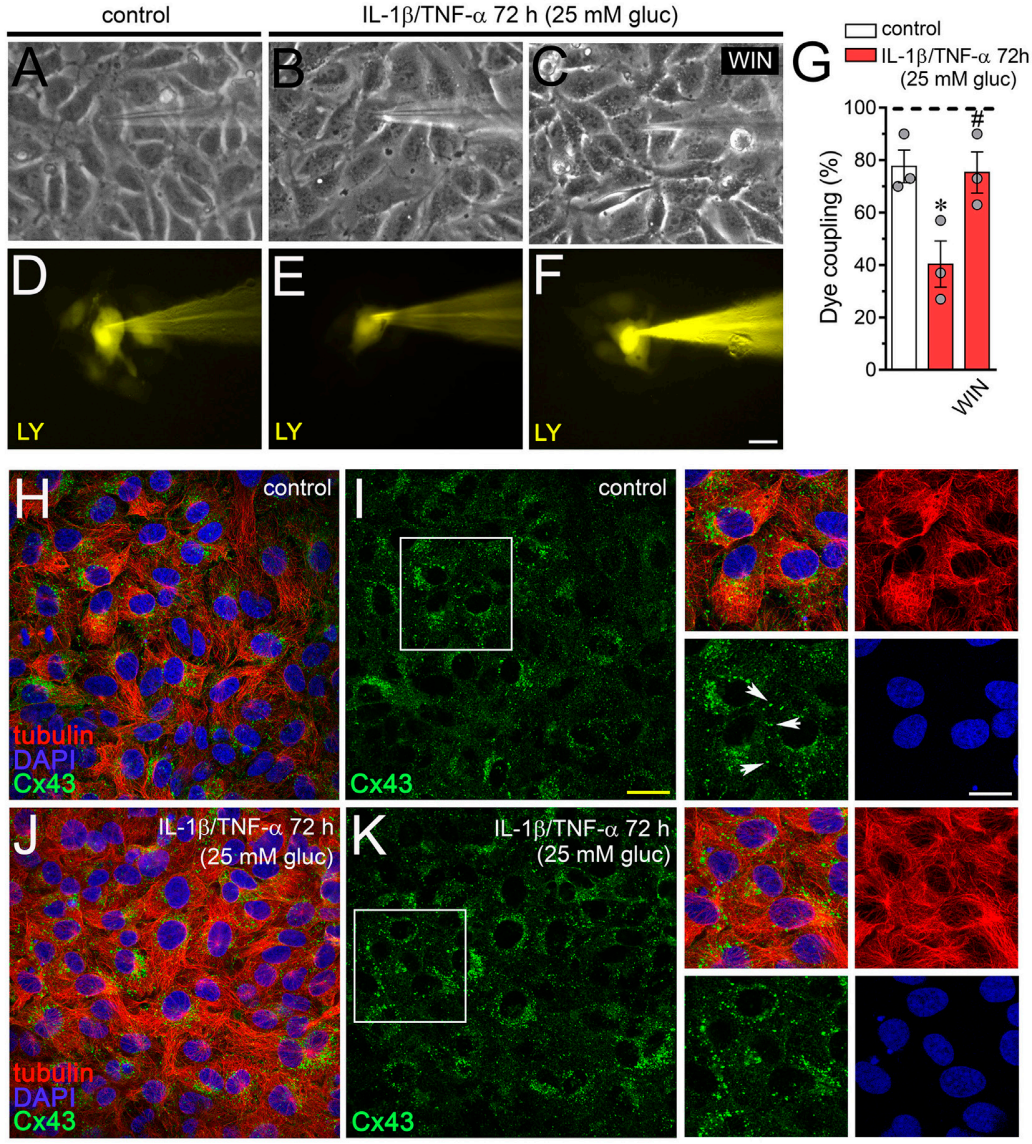
WIN prevents the high glucose and IL-1β/TNF-α-induced decrease in endothelial coupling through a mechanism that does not involve changes in connexin 43 (Cx43) distribution. **(A–F)** Representative fluorescence and phase contrast micrographs of Lucifer yellow (LY) transfer by EAhy cells treated for 72 h with 5 mM glucose [control **(A,D)**], 25 mM glucose, and IL-1β/TNF-α **(B,E)** alone or in combination with 5 µM WIN **(C,F)**. **(G)** Averaged data of dye coupling (percentage of injections that resulted in LY transfer) of EAhy cells treated for 72 h with 5 mM glucose (control, white bar), 25 mM glucose, and IL-1β/TNF-α (red bars) alone or in combination with 5 µM WIN. *p < 0.05, IL-1β/TNF-α and high glucose compared to control; ^#^*p* < 0.05, effect of each cannabinoid compared to the effect induced by IL-1β/TNF-α and high glucose (one-way analysis of variance followed by Dunnett’s *post hoc* test). Data were obtained from at least three independent experiments (see scatter dot plot) with four repeats each one (≥10 cells analyzed for each repeat). **(H–K)** Representative fluorescence images depicting Cx43 (green), tubulin (red), and DAPI (blue) immunolabeling of EAhy cells treated for 72 h with 5 mM glucose [control **(H,I)**] and 25 mM glucose plus IL-1β/TNF-α **(J,K)**. Insets: 1.7× magnification of the indicated area of panels **(I,K)**. White arrows indicate Cx43 labeling in cell–cell interfaces. Calibration bars: white = 35 µm, yellow = 60 µm, and green = 25 µm.

Endothelial-to-endothelial gap junctional communication is critical for the endothelium-derived hyperpolarization and concomitant vasodilation of the arteriolar smooth muscle (55). Given that increased Cx43 hemichannel opening induced by inflammatory conditions has been described to occur along with a rise in endothelial dye coupling (27), we evaluated if the endothelial gap junction coupling was altered upon treatment with IL-1β/TNF-α and high glucose. Under control conditions around ~80% of EAhy cells exhibited LY intercellular diffusion to neighboring cells (Figures 4A,D,G). Nonetheless, 72 h after treatment with IL-1β/TNF-α and high glucose intercellular dye transfer decreased by ~38% compared with control levels (Figures 4B,E,G). Equivalently, to the counteracting influence on IL-1β/TNF-α and high glucose-evoked Etd uptake, WIN entirely prevented the reduction in endothelial cell–cell coupling induced by IL-1β/TNF-α and high glucose (Figures 4C,F,G). Given that endocytosis of gap junctions from the plasma membrane is a process that might cause cellular uncoupling, we explored if IL-1β/TNF-α and high glucose-induced endothelial uncoupling was correlated with alterations in the cellular distribution of Cx43. In control EAhy cells, Cx43 was observed as fine to large granules scattered at cellular interfaces and perinuclear regions (Figures 4H,I) and comparable features were detected in those treated with IL-1β/TNF-α and high glucose (Figures 4J,K) or plus 5 µM WIN (Figures S1B–D in Supplementary Material). These findings indicate that IL-1β/TNF-α and high glucose-induced cell-to-cell uncoupling may depend on a mechanism implicating the closure and/or decreased permeability of Cx43 gap junctions rather than withdrawal from the apposition membranes.

### IL-1β/TNF-α Plus High Glucose Promotes the Cx43 Hemichannel-Induced Release of ATP From Endothelial Cells: Counteracting Action by WIN

**Figure 5:**
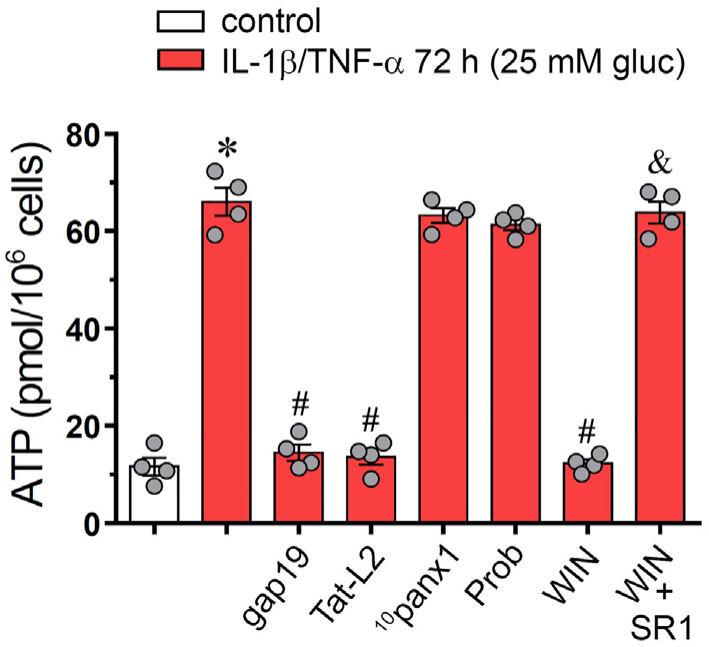
WIN mitigates the connexin 43 (Cx43) hemichannel-dependent release of ATP evoked by high glucose and IL-1β/TNF-α in endothelial cells. Averaged data of ATP release from EAhy cells treated for 72 h with 5 mM glucose (control, white bar), 25 mM glucose, and IL-1β/TNF-α (black bars) alone or in combination with the following agents: 100 µM gap19, 100 µM Tat-L2, 100 µM ^10^panx1, 500 µM probenecid (Prob), 5 µM, WIN and/or 5 µM SR-141716A (SR1). *p < 0.05, IL-1β/TNF-α and high glucose compared to control; ^#^*p* < 0.05, effect of each agent compared to the effect induced by IL-1β/TNF-α and high glucose; ^&^*p* < 0.05, effect of each cannabinoid receptor antagonist compared to the effect of the respective cannabinoid (one-way analysis of variance followed by Tukey’s *post hoc* test). Data were obtained from at least three independent experiments (see scatter dot plot) with four repeats each one.

Endothelial cells subjected to inflammatory conditions exhibit elevated release of ATP *via* the opening of Cx43 hemichannels (27, 56), a major signal involved in leukocyte recruitment and vascular inflammation (57). Given that P2X_7_ and P2Y_1_ receptors are involved in the Etd uptake evoked by IL-1β/TNF-α and high glucose in EAhy cells (Figure 2A), we evaluated whether this treatment could also impact the release of ATP *via* Cx43 hemichannels. IL-1β/TNF-α and high glucose strongly enhanced the release of ATP by ~6-folds compared to control conditions (Figure 5). Importantly, gap19 and Tat-L2, but not ^10^panx1 or probenecid, prominently reduced to control values the extracellular ATP concentration of cells treated with IL-1β/TNF-α and high glucose-induced release of ATP (from ~68 to ~13 and ~12 pmol/10^6^ cells, respectively) (Figure 5). These findings indicate that IL-1β/TNF-α and high glucose elevate the release of ATP in a Cx43 hemichannel-dependent form in EAhy endothelail cells. In this context, we tested the probable counteracting influence of WIN on this response. We observed that 1 h pretreatment and co-incubation with 5 µM WIN drastically reduced the IL-1β/TNF-α and high glucose-mediated release of ATP (from ~68 to ~11 pmol/10^6^ cells) (Figure 5). Interestingly, WIN failed in decreasing the ATP release in EAhy cells pre-incubated with 10 µM SR1 antagonist. Altogether, these findings support that CB_1_ receptors are the main contributors to the WIN-mediated inhibition of Cx43 hemichannel-dependent release of ATP evoked by IL-1β/TNF-α and high glucose in endothelial cells.

### IL-1β/TNF-α and High Glucose-Induced Changes in ATP-Dependent Ca^2+^ Dynamics Are Prevented by WIN in Endothelial Cells

**Figure 6:**
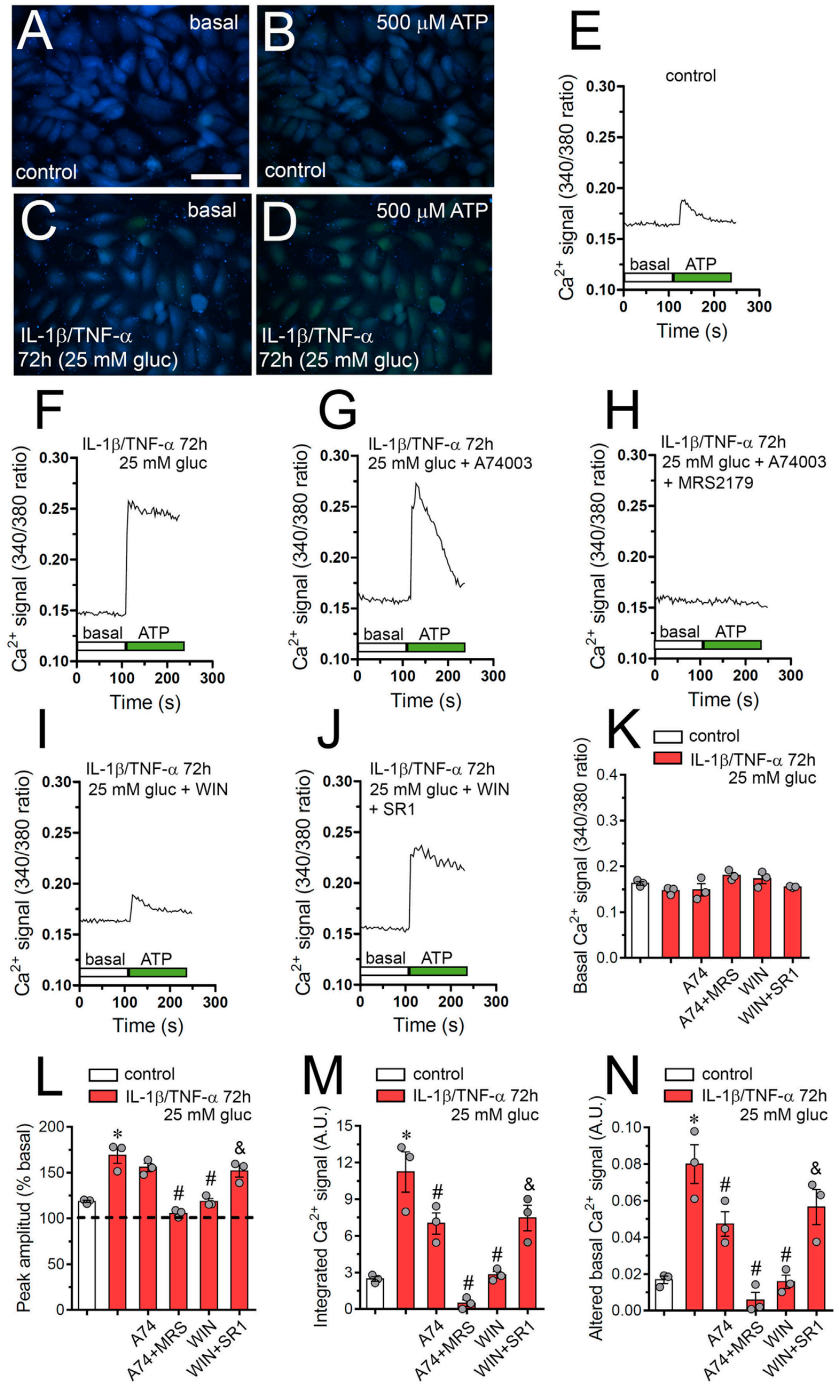
High glucose and IL-1β/TNF-α enhance ATP-dependent Ca^2+^ dynamics in endothelial cells: prevention by WIN. **(A–D)** Representative photomicrographs of basal **(A,C)** or 500 µM ATP-induced **(B,D)** Ca^2+^ signal denoted as Fura-2 ratio (340/380 nm excitation) of EAhy cells treated for 72 h with 5 mM glucose [control **(A,B)**] or 25 mM glucose and IL-1β/TNF-α **(C,D)**. Calibration bar: 180 µm. **(E–J)** Representative plots of relative changes in [Ca^2+^]_i_ signal over time induced by 500 µM ATP (gray vertical line) in EAhy cells treated for 72 h with 5 mM glucose [control **(E)**], 25 mM glucose, and IL-1β/TNF-α **(F)** alone or in combination with the following agents: 10 µM A740003 **(G)**, 10 µM A740003 plus 10 µM MRS2179 **(H)**, 5 µM, WIN **(I)** and 5 µM, WIN plus 5 µM SR-141716A [SR1 **(J)**]. **(K)** Averaged data of basal Fura-2 ratio by EAhy cells treated for 72 h with 5 mM glucose (control, white bar), 25 mM glucose, and IL-1β/TNF-α (red bars) alone or in combination with the following agents: 10 µM A740003 (A74), 10 µM A740003 plus 10 µM MRS2179 (A74 + MRS), 5 µM WIN (WIN) and 5 µM, WIN plus 5 µM SR-141716A (WIN + SR1). **(L–N)** Averaged data of ATP-induced peak amplitude normalized to basal Fura-2 ratio **(L)**, integrated ATP-induced Fura-2 ratio response **(M)**, and altered basal Fura-2 ratio **(N)** of EAhy cells treated for 72 h with 5 mM glucose (control, white bar), 25 mM glucose, and IL-1β/TNF-α (red bars) alone or in combination with the following agents: 10 µM A740003 (A74), 10 µM A740003 plus 10 µM MRS2179 (A74 + MRS), 5 µM WIN (WIN) and 5 µM, WIN plus 5 µM SR-141716A (WIN + SR1). *p < 0.05, IL-1β/TNF-α and high glucose compared to control; ^#^*p* < 0.05, effect of each pharmacological agent compared to the effect induced by IL-1β/TNF-α and high glucose; ^&^*p* < 0.05, effect of each cannabinoid receptor antagonist compared to the effect of the respective cannabinoid (one-way analysis of variance followed by Tukey’s *post hoc* test). Data were obtained from at least three independent experiments (see scatter dot plot) with four repeats each one (≥35 cells analyzed for each repeat).

Although cytoplasmic Ca^2+^ is crucial for proper endothelial barrier permeability and remodeling, its abnormal signaling during inflammatory conditions could lead to multiple vascular diseases (58, 59). Relevantly, both endothelial [Ca^2+^] signaling and hemichannel functional state are interdependent processes that may be enhanced during pathological conditions (60). In this context and because intracellular BAPTA greatly blunted IL-1β/TNF-α and high glucose-mediated Etd uptake (Figure 1A), we investigated if this condition could modulate the basal Ca^2+^ signal in EAhy cells. As indicated by the assessment of Fura-2 ratio (340/380), IL-1β/TNF-α and high glucose-stimulated EAhy cells showed basal Ca^2+^ signal that was similar to control conditions (Figures 6A,C,K). Despite that IL-1β/TNF-α and high glucose fail in modulate basal Ca^2+^ signal, these data do not rule out whether this condition affects the Ca^2+^ signal responses evoked by autocrine/paracrine signals, including ATP. With this in mind, we also studied the impact of IL-1β/TNF-α and high glucose on ATP-mediated Ca^2+^ signaling, as this transmitter can be released through Cx43 hemichannels from EAhy cells (Figure 6). Under control conditions, treatment with 500 µM ATP caused a rapid Ca^2+^ signal response with a small peak amplitude (Figures 6A,B,E,L). However, high glucose induced a sustained ATP-dependent Ca^2+^ signal response with a peak amplitude ~4-fold higher than that of control conditions (Figures 6C,D,F,L). This phenomenon was accompanied of a ~4- and ~4.5-fold increment in the integrated ATP-dependent Ca^2+^ signal response (Figure 6M) and the remaining difference between final and initial basal Ca^2+^ signal (Figure 6N), respectively.

In endothelial cells, ATP-mediated [Ca^2+^]_i_ responses involve different purinergic receptors, including P2X_7_ and P2Y_1_ receptors (61, 62), both being implicated in the hemichannel opening triggered by IL-1β/TNF-α and high glucose (Figure 2A). Notably, blockade of P2X_7_ receptors with 10 µM A740003 completely suppressed the sustained response pattern of ATP-mediated Ca^2+^ signal in IL-1β/TNF-α and high glucose-stimulated EAhy cells (Figure 6G). In addition, A740003 partially inhibited the integrated and remaining basal ATP-dependent Ca^2+^ signal responses (Figures 6M,N), but did not affect the peak amplitude induced by IL-1β/TNF-α and high glucose (Figure 6L). Notably, simultaneous inhibition of P2X_7_ and P2Y_1_ receptors with 10 µM A740003 and 10 µM MRS2179, respectively, completely blunted the ATP-dependent Ca^2+^ signal in IL-1β/TNF-α and high glucose-stimulated EAhy cells (Figure 6H). The latter was paralleled with a total suppression of the IL-1β/TNF-α and high glucose-induced increase of the Ca^2+^ signal evoked by ATP (Figures 6L–N). These findings indicate that upon ATP exposure, the transient peak in [Ca^2+^]_i_ signal recorded in IL-1β/TNF-α and high glucose-stimulated EAhy cells, likely came from Ca^2+^ released from intracellular stores due to stimulation of P2Y_1_ and IP_3_ receptors, whereas the following sustained Ca^2+^ signal could involve the participation of P2X_7_ receptors. Interestingly, IL-1β/TNF-α and high glucose-stimulated EAhy cells showed ATP-mediated Ca^2+^ signals similar to those recorded in control conditions when they were pre-treated with 10 µM WIN (Figures 6I,L–N). Moreover, WIN-induced prevention of ATP-induced Ca^2+^ signal did not occur in IL-1β/TNF-α and high glucose-stimulated EAhy cells co-incubated with the CB_1_ receptor antagonist SR1 (Figures 6J,L–N). The above data support that CB_1_ receptors are responsible of the WIN-mediated prevention in the disturbing actions of IL-1β/TNF-α and high glucose on the dynamics of ATP-mediated Ca^2+^ signals in endothelial cells.

### WIN and Blockers of Cx43 Hemichannels Prevent the NO Production of Endothelial Cells Treated With IL-1β/TNF-α and High Glucose

**Figure 7:**
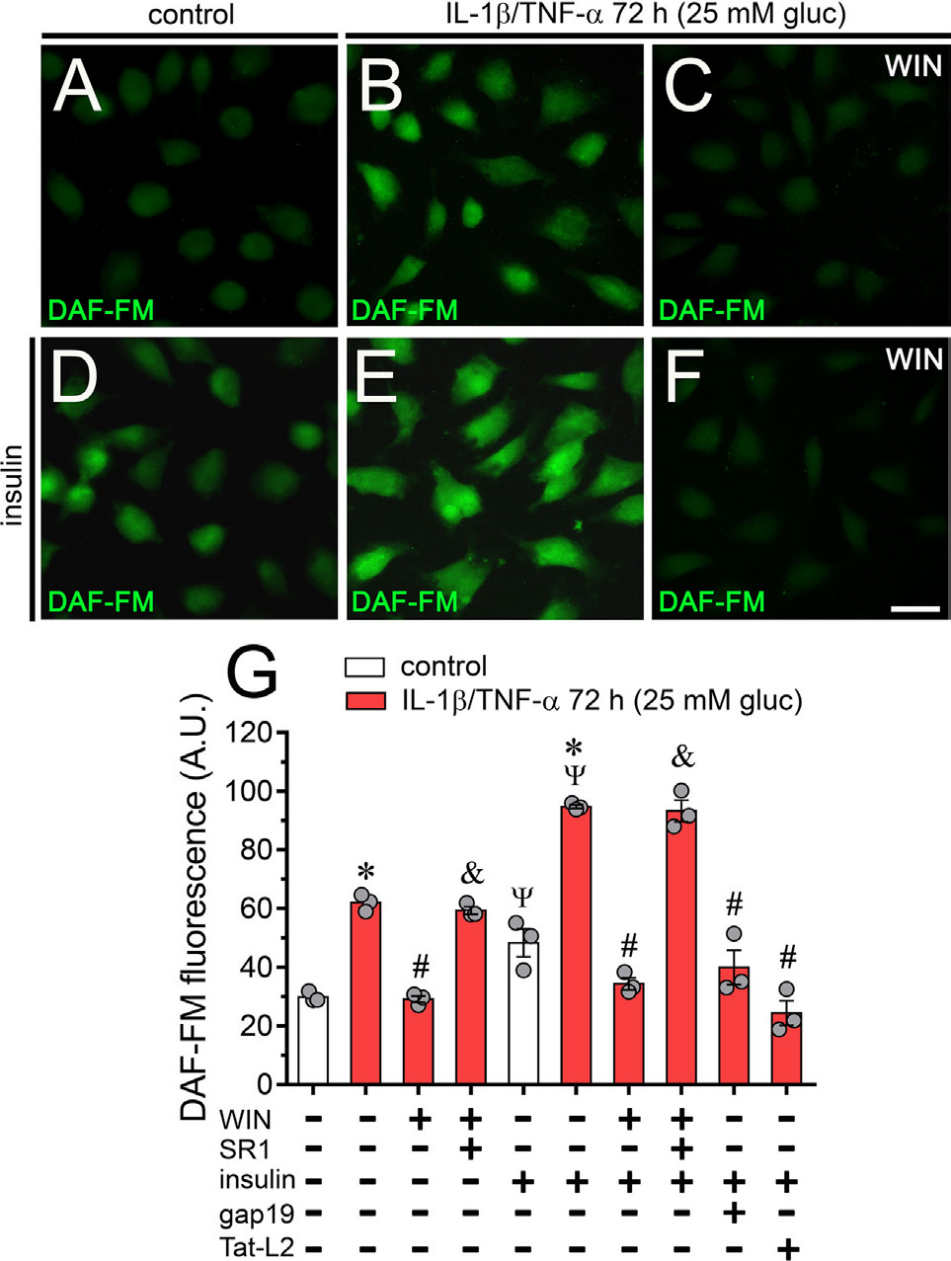
High glucose and IL-1β/TNF-α increase basal and insulin-induced production of nitric oxide (NO) in endothelial cells: prevention by WIN and connexin 43 (Cx43) hemichannel blockers. **(A–F)** Representative fluorescence micrographs of basal **(A–C)** or 1 µM insulin-induced **(D–F)** production of NO (DAF-FM, green and pseudo-colored scale) of EAhy cells treated for 72 h with 5 mM glucose [control **(A,D)**], 25 mM glucose, and IL-1β/TNF-α **(B,E)** alone or in combination with 5 µM WIN **(C,F)**. **(G)** Average of DAF fluorescence by EAhy cells treated for 72 h with 5 mM glucose (control; white bars), 25 mM glucose and IL-1β/TNF-α (red bars) alone or with different combinations of the following compounds: 5 µM WIN (WIN), 5 µM SR-141716A (SR1), 1 µM insulin, 100 µM gap19, and 100 µM Tat-L2. *p < 0.05, IL-1β/TNF-α and high glucose compared to control; ^#^*p* < 0.05, effect of each compound compared to the effect induced by IL-1β/TNF-α and high glucose; ^&^*p* < 0.05, effect of each cannabinoid receptor antagonist compared to the effect of the respective cannabinoid; ^Ψ^*p* < 0.05, effect of insulin compared to the respective control (one-way analysis of variance followed by Tukey’s *post hoc* test). Data were obtained from at least three independent experiments (see scatter dot plot) with four repeats each one (≥35 cells analyzed for each repeat). Calibration bar = 40 µm.

Altered iNOS-derived NO production has been involved in the beginning of acute and chronic inflammatory conditions associated with diverse diseases, including arthritis, sepsis, ischemia/reperfusion, diabetes, and atherosclerosis (63). Because LN-6, a specific iNOS blocker, strongly blunted the IL-1β/TNF-α and high glucose-mediated Etd uptake in EAhy cells (Figure 2A), we investigated whether Cx43 hemichannels also modulate NO production. DAF fluorescence experiments indicated that IL-1β/TNF-α and high glucose-treated EAhy cells exhibited a ~2-fold increase in basal NO amount compared with control values (Figures 7A,B,G). Interestingly, treatment with 5 µM WIN fully prevented the IL-1β/TNF-α and high glucose-induced increase in NO production, the latter response being dependent on CB_1_ receptors as SR1 abolished the counteracting action of WIN (Figures 7C,G). Insulin is a well-known inducer of NO production in normal endothelial cells, however, under inflammatory conditions, the insulin-mediated production of NO is impaired (64). In this context, we evaluated whether IL-1β/TNF-α and high glucose could disturb the insulin-mediated production of NO. As expected, 30 min treatment with 1 µM insulin induced a ~75% increase in NO levels in control EAhy cells (Figures 7D,G). Remarkably, IL-1β/TNF-α and high glucose increased in ~1-fold the insulin-mediated production of NO (Figures 7E,G), a response that was completely prevented by 5 µM WIN (Figures 7F,G). Supporting the involvement of CB_1_ receptors in the latter phenomenon, the counteracting influence of WIN on the insulin-mediated NO production did not occur in EAhy cells stimulated with IL-1β/TNF-α and high glucose plus co-incubation with SR1 (Figure 7G). Finally, we found that 100 µM gap19 or 100 µM Tat-L2 co-applied along with IL-1β/TNF-α and high glucose, fully suppressed the IL-1β/TNF-α and high glucose-mediated potentiation in NO production induced by insulin, turning NO levels to control values (Figure 7G). Altogether, these results support that Cx43 hemichannels serve as a crucial step in the modulatory actions evoked by IL-1β/TNF-α and high glucose on insulin-mediated production of NO in endothelial cells.

## Discussion

Here, we demonstrated for the first time that high glucose concentrations elevate the Cx43 hemichannel activity in cultured endothelial cells. A mixture of IL-1β and TNF-α, two pro-inflammatory cytokines that open hemichannels in different cell types (19, 20, 22), enhanced this phenomenon. Furthermore, IL-1β/TNF-α and high glucose-induced Cx43 hemichannel activity was associated with ATP release and activation of p38 MAPK, iNOS, COX_2_, PGE_2_ receptor EP_1_, and P2X_7_/P2Y_1_ receptors. In addition, we describe that a synthetic CB: WIN, and two endogenous CBs; 2-AG and Meth; counteract the IL-1β/TNF-α and high glucose-mediated Cx43 hemichannel opening and subsequent ATP release. These CBs also counteract diverse cell endothelial alterations evoked by IL-1β/TNF-α plus high glucose, including the increase in ATP-mediated Ca^2+^ signals and NO production.

As assayed by Etd uptake, we demonstrated that 45 mM glucose increments by itself the activity of Cx43 hemichannels in EAhy cells, whereas 25 mM glucose did it only in combination with the mixture of IL-1β and TNF-α. In fact, two specific mimetic peptides known to reduce Cx43 hemichannel opening (Tat-L2 and gap19), but not their inactive forms, significantly inhibited the IL-1β/TNF-α and high glucose-evoked Etd uptake. In addition, the latter effect did not occur in EAhy cells stimulated with siRNAs that downregulated Cx43. All these data indicate that IL-1β/TNF-α and high glucose specifically elevate the opening of Cx43 hemichannels in EAhy endothelial cells.

How do IL-1β/TNF-α and high glucose induce Cx43 hemichannel activity in EAhy endothelial cells? Prior research has determined that IL-1β and TNF-α or high glucose augment the functional state of Cx43 hemichannels *via* the p38 MAPK pathway and subsequent NO-mediated S-nitrosylation of Cx43 (20, 37, 39). Moreover, COX and PGE_2_ receptor EP_1_ stimulation is necessary for the long-lasting Cx43 hemichannel activity elicited during inflammatory conditions (38). Here, by using a battery of selective inhibitors, we have shown that the IL-1β/TNF-α and high glucose-induced Cx43 hemichannel opening comprises the activation of both p38 MAPK and iNOS, as well as the stimulation of PGE_2_ receptor EP_1_. In addition, IL-1β/TNF-α and high glucose raised the production of NO in EAhy cells (see below). These findings are consistent with the fact that NO stimulates COXs and the subsequent production of PGE_2_ (42). The latter prostaglandin is essential for [Ca^2+^]_i_ elevations evoked by EP_1_ receptors (44), which are highly expressed in endothelial cells (65).

**Figure 8:**
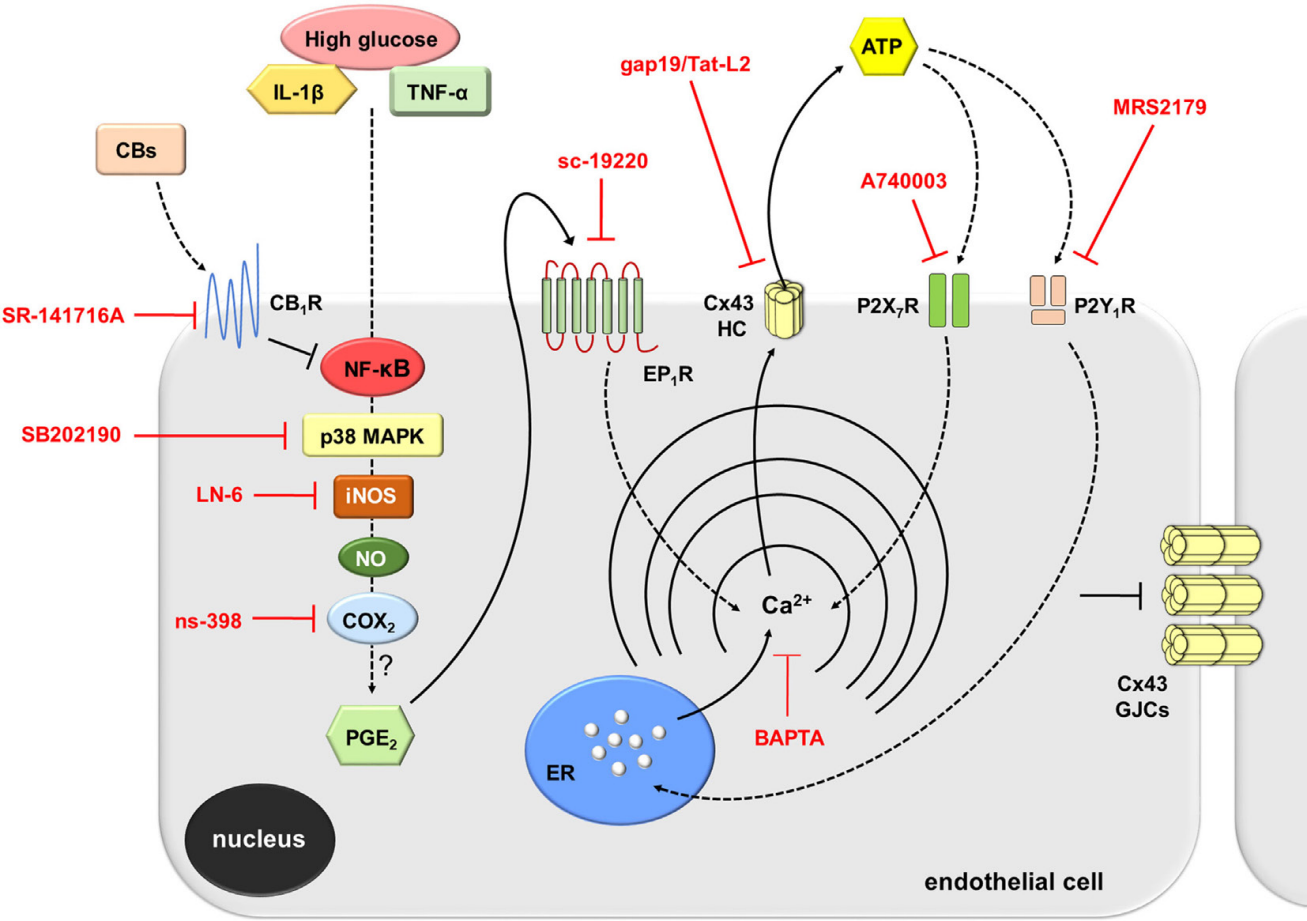
Schematic diagram showing the possible pathways involved in the activation of connexin 43 (Cx43) hemichannels evoked by high glucose and IL-1β/TNF-α in endothelial cells. Upon stimulation with high glucose and IL-1β/TNF-α, endothelial cells respond with intracellular signal transduction that possibly involve NF-κβ signaling associated with p38 MAPK and inducible NO synthase (iNOS) activation, nitric oxide (NO) production, and further stimulation of COX_2_. The latter likely induce the production of PGE_2_, which acting on EP_1_ metabotropic receptor promotes the release of Ca^2+^ from intracellular stores. Rise in [Ca^2+^]_i_, is a known condition that causes opening of Cx43 hemichannels enabling the release of ATP. ATP released *via* Cx43 hemichannels may activate P2X_7_ receptors, and its degradation to ADP may stimulate P2Y_1_ receptors. These events trigger the influx of extracellular Ca^2+^ and activation of IP_3_ receptors and further release of Ca^2+^ stored in the endoplasmic reticulum. The later induces an unknown self-perpetuating mechanism (see Discussion), in which high levels of [Ca^2+^]_i_ could reactivate iNOS, COX_2_, EP_1_ metabotropic receptors, and Cx43 hemichannels (not depicted). On the other hand, cannabinoids (CBs) acting on CB_1_Rs possibly counteract the NF-κβ-dependent activation of the above-mentioned pathways. This response results in the inhibition of p38 MAPK and NO production, as well as the consequent reduction ATP release through Cx43 hemichannels. In parallel, activation of CB_1_Rs may neutralize the reduction in gap junction communication evoked by high glucose and IL-1β/TNF-α. Main inhibitors used throughout this study are shown in red.

Multiple studies argue that pro-inflammatory cytokines or high glucose may contribute to a chronic activation of endothelial cells and thereby a long-term production of key “danger” signals, such as ATP (66–68), which is involved with vascular inflammation (62). In this context, two findings reveal that ATP signaling is fundamental in the opening of endothelial Cx43 hemichannels evoked by IL-1β/TNF-α and high glucose. First, we detected that blockade of both P2X_7_ and P2Y_1_ receptors partially abrogated the IL-1β/TNF-α and high glucose-induced Cx43 hemichannel activity. Second, the activity of Cx43 hemichannels was linked to the release of ATP in IL-1β/TNF-α and high glucose-stimulated EAhy cells. In conformity with this study, recent findings have elucidated that ATP elicits its own release *via* hemichannels and further stimulation of purinergic receptors (8, 38, 39). Here, autocrine/paracrine release of ATP seems to govern Cx43 hemichannel activity as an alternative mechanism to that caused by p38 MAPK and NO production (Figure 8). The activity of Cx43 hemichannels could take place upon elevations in [Ca^2+^]_i_ caused by activation of P2Y_1_ or P2X_7_ receptors (8, 38, 39). Accordingly, prior evidence have described that a moderate rise in [Ca^2+^]_i_ (>500 nM) increase the open probability of Cx43 hemichannel opening (6, 34, 45). In agreement with this evidence, we detected that chelation of [Ca^2+^]_i_ with BAPTA totally blunted the IL-1β/TNF-α and high glucose-induced Etd uptake in EAhy cells. In this scenario, endothelial Cx43 hemichannels could participate directly in the release of ATP, as they are permeable to this compound (69). Alternatively, because these channels are conduits for Ca^2+^ (70), they indirectly may contribute to perpetuate [Ca^2+^]_i_-dependent pathways associated with ATP release (e.g., exocytosis) (Figure 8). The intensity of this response might impact the outcome of the inflammation. In that regard, it has been demonstrated that opening of Cx43 hemichannels could lead to preconditioning (71) as well as to cell death (21).

Past research has established that plant-derived, synthetic, and endogenous CBs may provide protective actions against several cardiovascular pathologies, including ventricular arrhythmias (72) and cardiomyopathies (73). In fact, CBs diminish endothelial dysfunction by inhibiting the production of inflammatory mediators (e.g., free radical and cytokines) and their signaling pathways (e.g., NF-κβ) (53, 74). However, whether endothelial hemichannels are part of the targets involved in the anti-inflammatory actions of CBs remained unknown. Here, we observed that WIN, 2-AG, and Meth completely suppressed the Cx43 hemichannel-mediated Etd uptake induced by IL-1β/TNF-α and high glucose in EAhy cells. These preventive actions were completely neutralized by the CB_1_ receptor antagonist SR1, which is in according with the participation of CB_1_ receptors in Cx43 hemichannel opening (48, 50), as well as their demonstrated expression and function in endothelial cells (52, 75). Interestingly, WIN fully reduced not only the IL-1β/TNF-α and high glucose-induced Etd uptake but also significantly prevented the release of ATP triggered by these pro-inflammatory conditions. Similar inhibitory responses on Cx43 hemichannel-dependent ATP release have been observed for CBs in activated astrocytes (50). Other mechanism of hemichannel modulation different of that resulting from covalent modifications (e.g., phosphorylation and/or S-nitrosylation) is the trafficking of hemichannels to non-junctional membranes. In this study, we demonstrated that WIN fully abrogated the IL-1β/TNF-α and high glucose-induced augment in plasma membrane levels of Cx43, revealing that alterations in surface protein expression are possibly sufficient to account for the regulation of hemichannel activity triggered by IL-1β/TNF-α and high glucose or CBs in EAhy cells. It is important to mention that pharmacotherapy involving CBs is still under intense debate. The latter is mainly due to the negative side effects that CBs may exert on the nervous system and peripheral glucose metabolism (76, 77) most likely due to their low affinity to the molecular targets. Future studies will elucidate whether or not targeting specifically endothelial cells with CB receptor agonists could counteract endothelial dysfunction *in vivo*.

Multiple lines of work point out that hemichannels and gap junctions are contrarily modulated by inflammatory agents (78). In agreement with those observations, we noted that in addition to elevate endothelial Cx43 hemichannel activity, IL-1β/TNF-α and high glucose suppressed the cell-to-cell gap junctional communication in EAhy cells, as measured by intercellular LY diffusion. Relevantly, WIN fully prevented the IL-1β/TNF-α and high glucose-induced reduction in endothelial coupling *via* the activation of CB1 receptors. As deducted from western blot analysis, the modulation of dye coupling triggered by IL-1β/TNF-α and high glucose or WIN could be in part attributed to changes in Cx43 amount, namely, total reduction or increment, respectively. Moreover, immunofluorescence labeling showed no differences in the distribution of Cx43 in EAhy cells treated with IL-1β/TNF-α and high glucose alone or plus WIN, indicating that endocytosis or degradation of gap junctions do not account for the regulation of endothelial coupling.

Prior findings in diverse cell types, including endothelial cells, have revealed that ATP produces a biphasic [Ca^2+^]_i_ response: the release of stored Ca^2+^ (first spike) and Ca^2+^ influx from the extracellular medium (sustained response) (61, 79). The first spike in ATP-elicited [Ca^2+^]_i_ response depends on P2Y receptors, while the second sustained event take place due to P2X receptors. Here, we noticed that upon acute stimulation with ATP, control EAhy cells displayed a small Ca^2+^ signal peak that returned rapidly to control values. In contrast, IL-1β/TNF-α and high glucose-treated EAhy cells showed increased ATP-induced Ca^2+^ signals compared to control, particularly, in terms of peak amplitude, integrated area, and sustained signal. Notably, in these conditions the initial Ca^2+^ signal peak was inhibited by MRS2179, but not by P2X_7_ receptor blockers, suggesting the implication of metabotropic P2Y_1_ receptors. Given that ADP is the major ligand for P2Y_1_ receptors, and because they participate in endothelial Ca^2+^ dynamics (80), in our studies, ADP produced from ATP conversion possibly generated the P2Y_1_-mediated changes in [Ca^2+^]_i_ elicited by acute ATP application. On the other hand, the ATP-induced sustained Ca^2+^ signal detected in IL-1β/TNF-α and high glucose-stimulated EAhy cells was fully counteracted by blocking P2X_7_ receptors, indicating that influx of Ca^2+^ is also necessary for ATP-induced Ca^2+^ signal in EAhy cells. Interestingly, the above Ca^2+^ response associated with P2Y_1_/P2X_7_ receptors was completely inhibited by WIN-dependent activation of CB1 receptors in IL-1β/TNF-α and high glucose-stimulated EAhy cells. These data denote that ATP-mediated Ca^2+^ dynamics depend on the inflammatory profile of endothelial cells and can be antagonized by the anti-inflammatory actions of CBs (Figure 8). ATP released from endothelial cells could activate distant cells in a paracrine form, resulting in Ca^2+^ responses that may rely on the endothelial inflammatory profile. In this scenario, stimulation of purinergic receptors may be shut down due to diffusion of ATP to far-off areas in conjunction with desensitization of P2Y_1_ receptors and degradation of extracellular ATP *via* exonucleases.

In endothelial cells, NO can be produced from l-arginine in a reaction catalyzed by endothelial NO synthase (eNOS) and iNOS (36, 81). Yet despite both NOS isoforms catalyze the same biochemical reaction, eNOS and iNOS are very different enzymes, being the former involved in the constitutive NO production at nanomolar levels, whereas the latter generates micromolar amounts of NO only when stimulated (82). NO exerts important vasodilatory and protective effects on the vasculature (83). However, high NO production has been linked to the pathogenesis of chronic inflammatory diseases, including atherosclerosis (63). Relevant to this point, previous studies have revealed that pro-inflammatory conditions (e.g., high glucose) elicit the formation of endothelial NO (36, 84). In agreement with this information, we identify that IL-1β/TNF-α and high glucose clearly increase NO production in EAhy cells, which could be an alternative mechanism of hemichannel regulation through the S-nitrosylation of Cx43 (37).

Insulin is a direct-acting vasodilator in intact vessels (85) and has been described to induce the production of NO in normal endothelial cells (86). Nevertheless, endothelial cells subjected to pro-inflammatory conditions, such as high glucose and cytokine treatment, loss the ability to form NO (87, 88). As expected, in control EAhy cells, insulin promoted an evident augment in NO production. Surprisingly, in EAhy cells stimulated with IL-1β/TNF-α and high glucose, the insulin-mediated NO production was higher than that of control conditions, revealing that insulin sensitivity is not inhibited. This unexpected finding might occur by the degree of inflammation developed by EAhy cells under the pro-inflammatory treatment used. Perhaps the NO response to insulin treatment become reduced at later time points not analyzed in the present work or the application of more intense pro-inflammatory conditions is required to develop that outcome. As occurred with IL-1β/TNF-α and high glucose-induced changes in hemichannel opening, ATP release and [Ca^2+^]_i_ dynamics, the enhanced production of NO was greatly prevented by the activation of CB_1_ receptors with WIN.

High glucose and IL-1β/TNF-α are well established conditions that disturb vascular homeostasis through different cellular and molecular mechanisms (5). Here, we identify the function of endothelial Cx43 hemichannels as a new pathway affected by inflammatory mediators, revealing their possible implication in the pathogenesis of multiple vascular diseases. Supporting this idea, the increased production of NO caused by IL-1β/TNF-α and high glucose was completely impeded by blockade of endothelial Cx43 hemichannels. Furthermore, this study demonstrated that intracellular Ca^2+^ associated with COX_2_/EP_1_ receptor signaling and purinergic receptor activation—likely *via* ATP release—are crucial to maintain persistent opening of Cx43 hemichannels and possibly for preserving the p38 MAPK-dependent NO production observed in IL-1β/TNF-α and high glucose-stimulated endothelial cells. The above may reproduce a self-perpetuating mechanism, in which both NO or high [Ca^2+^]_i_ levels could reactivate Cx43 hemichannels (Figure 8). This phenomenon likely may lead to cell death, either by Ca^2+^ overload or through the reaction of NO with the superoxide anion, which yield peroxynitrite, a potent oxidant that alters DNA, lipids and proteins. We propose that reduction of hemichannel activity by CB agonists or selective hemichannel blockers might represent a strategy against the activation of deleterious pathways that trigger endothelial dysfunction and possibly cell damage evoked by high glucose and pro-inflammatory cytokines. The latter should favor the generation and design of novel CB agonists that could preserve their positive role without having side effects in general physiology.

## Ethics Statement

This study was carried out in accordance with the recommendations of the Animal Care Guidelines of the Research Ethic Committee from the Pontificia Universidad Católica de Chile. The protocol was approved by Research Ethic Committee from the Pontificia Universidad Católica de Chile.

## Author Contributions

Conceived, performed, and analyzed the experiments: JAO, JCS, VV, SC-D, GG, VL, CS, RG-G, BA, ED, and TM. Wrote and edited the manuscript: JAO, JCS, and VV. All authors read and approved the final manuscript.

## Conflict of Interest Statement

The authors declare that the research was conducted in the absence of any commercial or financial relationships that could be construed as a potential conflict of interest.

## Supplementary Material

Figure S1 Etd uptake induced by high glucose and IL-1β/TNF-α is not related to osmolarity changes, whereas high glucose/IL-1β/TNF-α plus WIN do not affect connexin 43 (Cx43) distribution in endothelial cells. **(A)** Averaged Etd uptake rate normalized with respect to control condition (dashed line) of EAhy cells treated for 72 h with 5 mM glucose and IL-1β/TNF-α alone or in combination with 20 or 40 mM sucrose or 20 or 40 mM mannitol. Data were obtained from three independent experiments (see scatter dot plot) with two repeats each one (≥35 cells analyzed for each repeat). **(B,C)** Representative fluorescence images depicting Cx43 (green), tubulin (red), and DAPI (blue) immunolabeling of EAhy cells treated for 72 h with 25 mM glucose plus IL-1β/TNF-α and 5 µM WIN. Insets: 1.7× magnification of the indicated area of panels **(C)**. Calibration bars: white = 35 µm, yellow = 60 µm, and green = 25 µm.

Figure S2 High glucose and IL-1β/TNF-α increase the activity of connexin 43 hemichannels and nitric oxide production in HUVEC endothelial cells. **(A)** Averaged Etd uptake rate normalized with control condition (dashed line) by HUVEC cells treated for 72 h with 25 mM glucose and IL-1β/TNF-α alone or in combination with the following blockers: 100 µM gap26, 100 µM ^10^panx1, 10 µM WIN or 5 µM WIN plus 5 µM SR-141716A (SR1). **p* < 0.05, IL-1β/TNF-α and high glucose compared to control; ^#^*p* < 0.05, effect of blockers compared IL-1β/TNF-α and high glucose. **(B)** Average of DAF fluorescence by HUVEC cells treated for 72 h with 5 mM glucose (control; white bars), 25 mM glucose and IL-1β/TNF-α (red bars) alone or with different combinations of the following compounds: 5 µM WIN (WIN), 5 µM SR-141716A (SR1), 1 µM insulin or 100 µM gap26. **p* < 0.05, IL-1β/TNF-α and high glucose compared to control; ^#^*p* < 0.05, effect of each compound compared to the effect induced by IL-1β/TNF-α and high glucose; ^&^*p* < 0.05, effect of each cannabinoid receptor antagonist compared to the effect of the respective cannabinoid; ^Ψ^*p* < 0.05, effect of insulin compared to the respective control (one-way analysis of variance followed by Tukey’s *post hoc* test). Data were obtained from three independent experiments (see scatter dot plot) with three repeats each one (≥35 cells analyzed for each repeat).
