# Supplementary figures and images for: Connexin 43 Hemichannel Activity Promoted by Pro-Inflammatory Cytokines and High Glucose Alters Endothelial Cell Function
Source: Front Immunol. 2018 Aug 15;9:1899. doi: 10.3389/fimmu.2018.01899 (PMC6104120; doi:10.3389/fimmu.2018.01899)

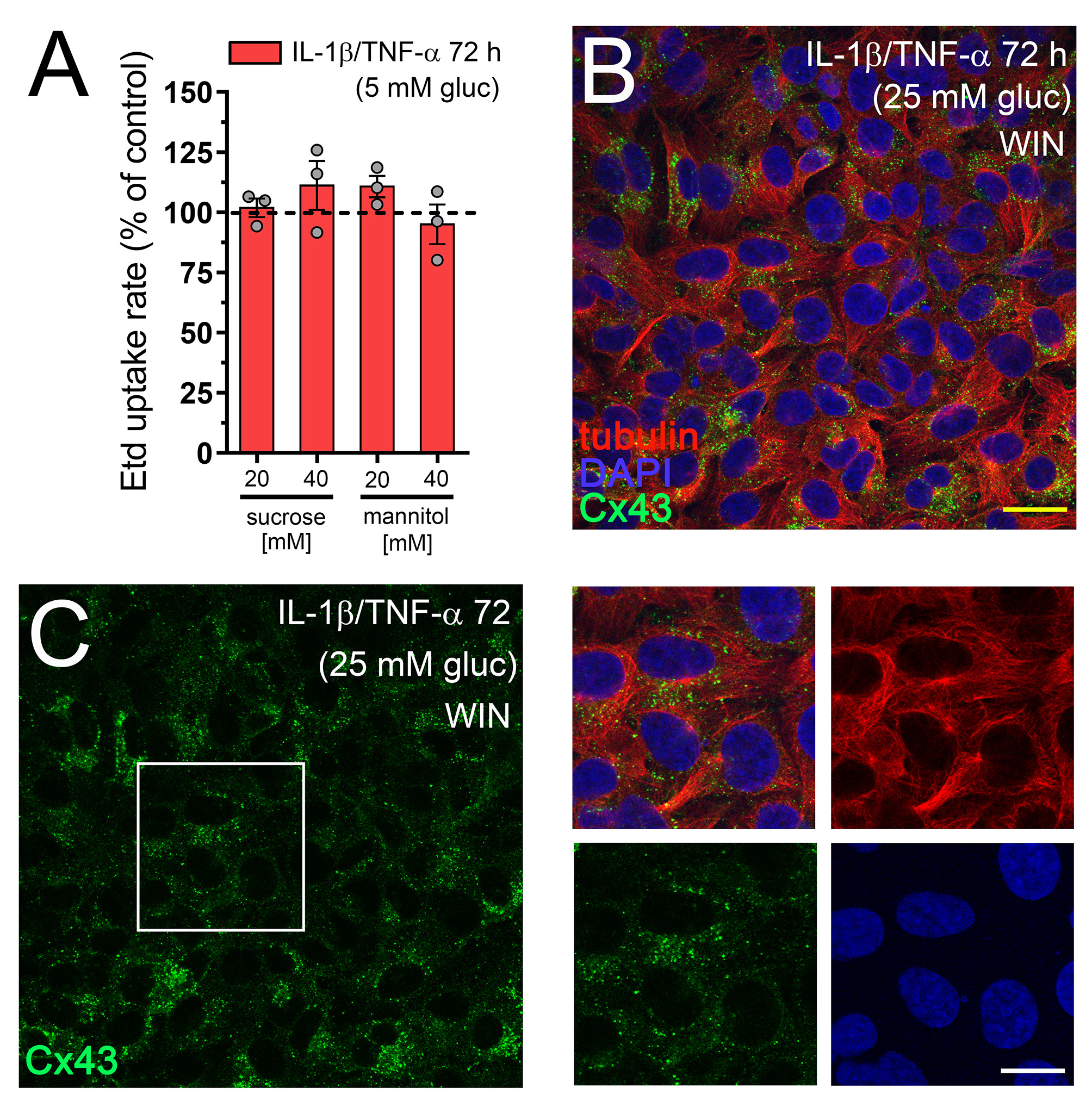

Supplement: Figure S1 — Etd uptake induced by high glucose and IL-1β/TNF-α is not related to osmolarity changes, whereas high glucose/IL-1β/TNF-α plus WIN do not affect connexin 43 (Cx43) distribution in endothelial cells. (A) Averaged Etd uptake rate normalized with respect to control condition (dashed line) of EAhy cells treated for 72 h with 5 mM glucose and IL-1β/TNF-α alone or in combination with 20 or 40 mM sucrose or 20 or 40 mM mannitol. Data were obtained from three independent experiments (see scatter dot plot) with two repeats each one (≥35 cells analyzed for each repeat). (B,C) Representative fluorescence images depicting Cx43 (green), tubulin (red), and DAPI (blue) immunolabeling of EAhy cells treated for 72 h with 25 mM glucose plus IL-1β/TNF-α and 5 µM WIN. Insets: 1.7× magnification of the indicated area of panels (C). Calibration bars: white = 35 µm, yellow = 60 µm, and green = 25 µm. [file image_1.jpeg]

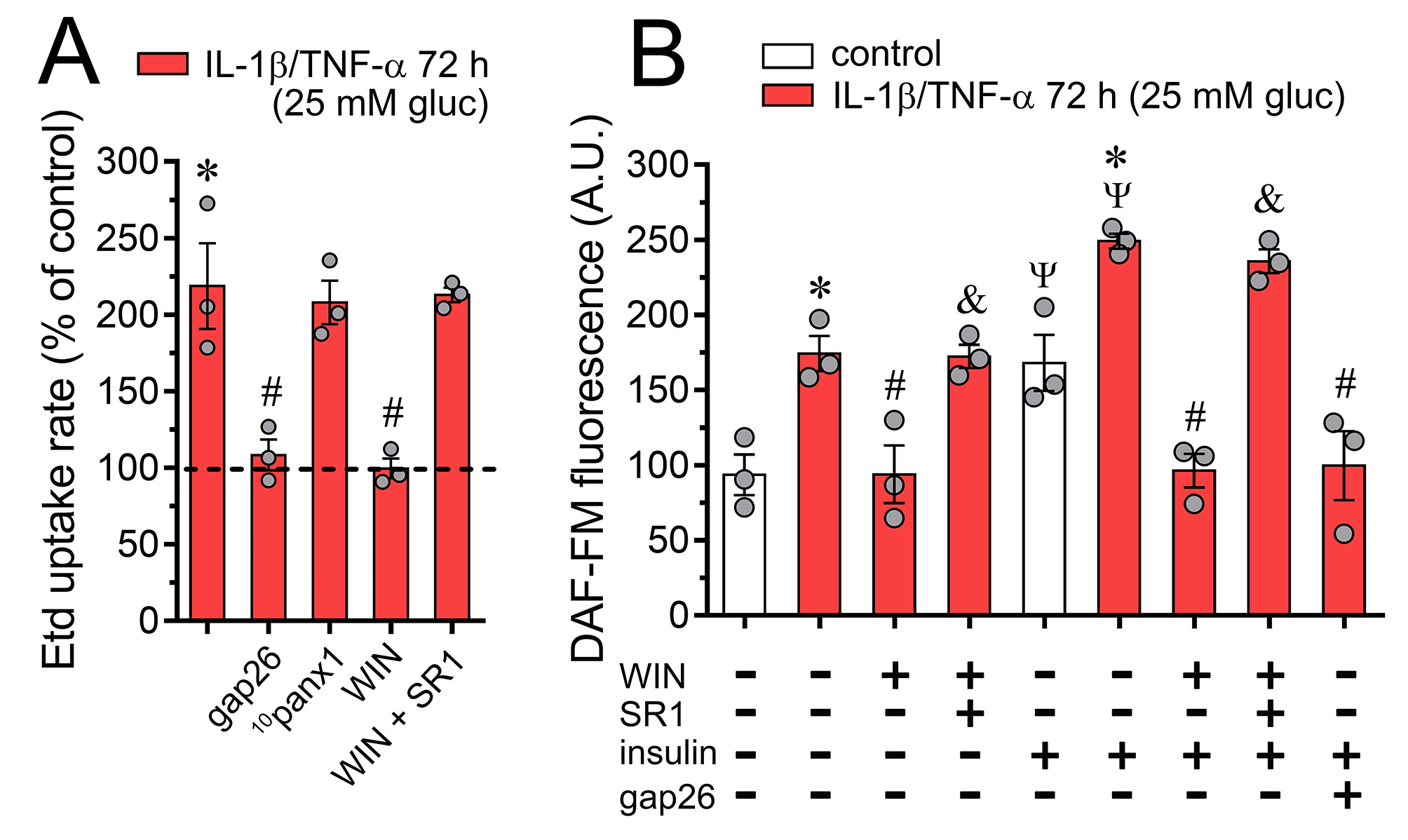

Supplement: Figure S2 — High glucose and IL-1β/TNF-α increase the activity of connexin 43 hemichannels and nitric oxide production in HUVEC endothelial cells. (A) Averaged Etd uptake rate normalized with control condition (dashed line) by HUVEC cells treated for 72 h with 25 mM glucose and IL-1β/TNF-α alone or in combination with the following blockers: 100 µM gap26, 100 µM 10panx1, 10 µM WIN or 5 µM WIN plus 5 µM SR-141716A (SR1). *p < 0.05, IL-1β/TNF-α and high glucose compared to control; #p < 0.05, effect of blockers compared IL-1β/TNF-α and high glucose. (B) Average of DAF fluorescence by HUVEC cells treated for 72 h with 5 mM glucose (control; white bars), 25 mM glucose and IL-1β/TNF-α (red bars) alone or with different combinations of the following compounds: 5 µM WIN (WIN), 5 µM SR-141716A (SR1), 1 µM insulin or 100 µM gap26. *p < 0.05, IL-1β/TNF-α and high glucose compared to control; #p < 0.05, effect of each compound compared to the effect induced by IL-1β/TNF-α and high glucose; &p < 0.05, effect of each cannabinoid receptor antagonist compared to the effect of the respective cannabinoid; Ψp < 0.05, effect of insulin compared to the respective control (one-way analysis of variance followed by Tukey’s post hoc test). Data were obtained from three independent experiments (see scatter dot plot) with three repeats each one (≥35 cells analyzed for each repeat). [file image_2.jpeg]
